# Variations on a theme: diversification of cuticular hydrocarbons in a clade of cactophilic *Drosophila*

**DOI:** 10.1186/1471-2148-11-179

**Published:** 2011-06-23

**Authors:** Cássia C de Oliveira, Maura H Manfrin, Fábio de M Sene, Larry L Jackson, William J Etges

**Affiliations:** 1 Program in Ecology and Evolutionary Biology, Department of Biological Sciences, SCEN 632, University of Arkansas, Fayetteville, AR 72701, USA; 2 School of Biological Sciences, 302 Manter Hall, University of Nebraska, Lincoln, NE 68588, USA; 3 Departamento de Biologia, Faculdade de Filosofia, Ciências e Letras de Ribeirão Preto, Universidade de São Paulo, Av. dos Bandeirantes, 3900 Ribeirão Preto-SP, 14040-901, Brazil; 4 Departamento de Genética, Faculdade de Medicina de Ribeirão Preto, Universidade de São Paulo, Av. dos Bandeirantes, 3900 Ribeirão Preto-SP, 14049-900, Brazil; 5 Chemistry and Biochemistry Department, Montana State University, Bozeman, MT 59717, USA

## Abstract

**Background:**

We characterized variation and chemical composition of epicuticular hydrocarbons (CHCs) in the seven species of the *Drosophila buzzatii *cluster with gas chromatography/mass spectrometry. Despite the critical role of CHCs in providing resistance to desiccation and involvement in communication, such as courtship behavior, mating, and aggregation, few studies have investigated how CHC profiles evolve within and between species in a phylogenetic context. We analyzed quantitative differences in CHC profiles in populations of the *D. buzzatii *species cluster in order to assess the concordance of CHC differentiation with species divergence.

**Results:**

Thirty-six CHC components were scored in single fly extracts with carbon chain lengths ranging from C_29 _to C_39_, including methyl-branched alkanes, *n*-alkenes, and alkadienes. Multivariate analysis of variance revealed that CHC amounts were significantly different among all species and canonical discriminant function (CDF) analysis resolved all species into distinct, non-overlapping groups. Significant intraspecific variation was found in different populations of *D. serido *suggesting that this taxon is comprised of at least two species. We summarized CHC variation using CDF analysis and mapped the first five CHC canonical variates (CVs) onto an independently derived *period *(*per*) gene + chromosome inversion + mtDNA COI gene for each sex. We found that the COI sequences were not phylogenetically informative due to introgression between some species, so only *per *+ inversion data were used. Positive phylogenetic signal was observed mainly for CV1 when parsimony methods and the test for serial independence (TFSI) were used. These results changed when no outgroup species were included in the analysis and phylogenetic signal was then observed for female CV3 and/or CV4 and male CV4 and CV5. Finally, removal of divergent populations of *D. serido *significantly increased the amount of phylogenetic signal as up to four out of five CVs then displayed positive phylogenetic signal.

**Conclusions:**

CHCs were conserved among species while quantitative differences in CHC profiles between populations and species were statistically significant. Most CHCs were species-, population-, and sex-specific. Mapping CHCs onto an independently derived phylogeny revealed that a significant portion of CHC variation was explained by species' systematic affinities indicating phylogenetic conservatism in the evolution of these hydrocarbon arrays, presumptive waterproofing compounds and courtship signals as in many other drosophilid species.

## Background

The nested hierarchical nature of species due to shared ancestry has been useful in comparative biology to assess relative rates of phenotypic evolution [1]. In a comprehensive comparative study, Blomberg et al. [2] showed that behavioral traits were more labile (weakly or uncorrelated with phylogeny) than body size, morphological, life-history, or physiological characters. Conversely, Wimberger and de Queiroz [3] found no significant difference in evolutionary lability between morphological and behavioral traits. Therefore, relative evolutionary rates of morphological and physiological vs. behavioral traits is still being debated [4,5], and resolution may depend on the kinds of traits studied and the degree of phylogenetic resolution of focal species groups.

Among arthropods, common species-specific phenotypes that influence organismal water balance and also serve as contact pheromones, particularly in insects, are cuticular waxes composed of hydrocarbons [6-12]. In *Drosophila*, epicuticular hydrocarbon (CHC) components are usually sex-specific, species-specific and sometimes geographically variable [7,13-18]. These molecules are integral to the waterproofing functions of the insect cuticle, providing resistance to desiccation and water loss [19-21]. Despite the involvement of CHCs with cuticular water flux, mate recognition, and in some cases reproductive isolation, little is known about the mechanisms responsible for their larger scale diversification because few studies have investigated how correlated CHC differences evolve in a phylogenetic context [reviewed in [22]]. Further, the nature of CHC variation can be both qualitative and quantitative [7,13,23]: CHC composition can be dynamic and change with age [24,25], is influenced by temperature [24], larval-rearing substrates [26,27], and members of the opposite sex [28-31] suggesting significant sources of variation that may inhibit attempts to map their evolution onto species/population phylogenies. Using groups of populations/species in various stages of divergence is essential if we are to gauge rates of evolution across a spectrum of genetic differences including the final stages of speciation [32]. This way, we can gauge which phenotypes evolve before others, and attempt to identify causal factors responsible for divergence and perhaps the formation of new species [33].

### Phylogeny of the *D. buzzatii *Cluster

**Figure 1:**
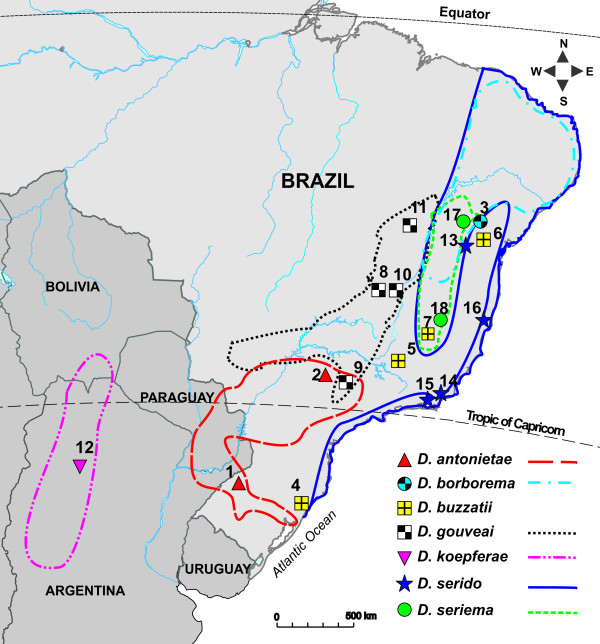
**Partial view of South American map showing the geographic distribution of the species in the *D. buzzatii *cluster**. The distribution of *D. buzzatii *is not marked because it is found in all areas where the other species occur. Numbers represent the localities of the eighteen populations/species used in the CHC analysis (see Table 1).

We analyzed evolution of quantitative differences in CHC profiles in a recently diverged species group of *Drosophila*, the *D. buzzatii *cluster, in order to assess phylogenetic influences on these species-specific epicuticular hydrocarbons. This monophyletic group of cactophilic *Drosophila *has been previously characterized in terms of its biogeography and ecology [reviewed in [34]]. The *D. buzzatii *cluster is part of the *mulleri *complex in the large *D. repleta *group, and consists of seven closely related species including *D. buzzatii *[35], *D. serido*, *D. borborema *[36], *D. koepferae *[37], *D. seriema *[38], *D. antonietae*, and *D. gouveai *[39]. Except for cosmopolitan *D. buzzatii*, the other species are endemic to South America with most distributed in and around Brazil (Figure 1). All of these species are cryptic, where species can only be identified morphologically using male genital characteristics [39].

**Figure 2:**
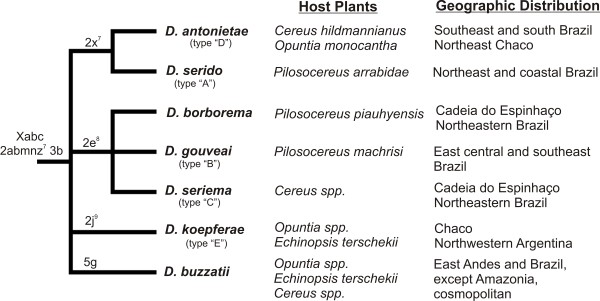
**Consensus phylogeny of the *D. buzzatii *species cluster based on chromosomal inversions and ecological/geographical affiliations for each species**. Male genitalia (aedeagus) types (A - E) for the species of the *D. buzzatii *cluster are labeled according to Silva and Sene [105]. *D. buzzatii *and *D. borborema *were not included in that classification because both species have aedeagi that were already well characterized and could be easily distinguishable from the other species. Chromosomal inversions, shown above the tree branches, are based on Ruiz et al. [41,53] and used together with *period *gene data to reconstruct the phylogeny (see Figures 5 and 6). Host plant use and geographic distributions are based on Manfrin and Sene [34], Benado et al. [106], Marín et al. [107] and Vilela [108].

Monophyly of the *D. buzzatii *cluster was first proposed on the basis of multiple chromosomal inversions [40], with four inversions unique to different species [41]. Despite being reliable phylogenetic markers [42,43], chromosomal inversions cannot resolve the relationships among some of these species, i.e. *D. borborema*, *D. gouveai *and *D. seriema*, as no inversions are unique to these taxa (Figure 2). Phylogenetic analysis of mtDNA cytochrome oxidase I (COI) sequences confirmed that these seven species form a monophyletic group [44,45]. However, within the cluster, not all populations of the same species were recovered in the same clade or shared the closest branches in the tree. While a mtDNA COI phylogeny partially agreed with the chromosome phylogeny, haplotype sharing among populations was observed suggesting secondary contact between *D. antonietae *and *D. gouveai *[34,44] making these COI data less than informative for character mapping. Recently, Franco et al. [46] proposed a phylogeny for the cluster based on the nuclear *period *(*per) *gene. This phylogeny also confirmed that the *D. buzzatii *cluster forms a monophyletic group and also resolved the relationships among populations of all species including *D. gouveai*, *D. borborema *and *D. seriema*.

### Ecology and Biogeography of the *D. buzzatii *Cluster

All species of the *D. buzzatii *cluster are cactophilic so their ranges are associated with the distributions of their host plants (Figure 2). *D. buzzatii *cluster species feed and breed exclusively in necrotic cactus tissues (rots) [41,47] and some species are oligophagic, while others appear to be more specialized (Figure 2). These species are distributed throughout the caatinga and Chaco morphoclimatic domains along a corridor of arid xeromorphic vegetation extending from the northeast to the southwest between the Amazonian and Atlantic rainforests of South America. Adjacent dry forests also include cacti, but as isolated populations. These isolates are thought to have resulted from repeated retractions and expansions of open vegetation during the Quaternary glacial and interglacial periods, respectively, affecting the differentiation and speciation of *D. buzzatii *cluster species [34,44]. Nested clade analysis of Brazilian *D. buzzatii *cluster species suggested that these species have been distributed across Brazil at least since the Mid-Pleistocene [48]. It is likely that these climatic alterations have promoted repeated waxing and waning of cactus populations in Brazil and elsewhere in South America.

Thus, the phylogeny, biogeography, and ecology of the *D. buzzatii *cluster should help us to understand phenotypic evolution among populations of these recently diverged species, some that can still hybridize in nature, and how sexually dimorphic and typically species-specific CHCs have evolved in these species. Therefore, we characterized the variation and chemical composition of CHCs in all seven species in the cluster so that we could uncover the role these compounds may play in desiccation resistance and as recognition signals within and between species. By mapping CHC variation onto a phylogeny of these species, we show that correlated groups of CHCs show discordant patterns of evolution with some CHCs showing significant phylogenetic signal and others evolving more rapidly.

## Methods

### Origin and Maintenance of Fly Stocks

All populations and species were collected in the wild using fruit baits (Figure 1, Table 1) and were maintained in the Departamento de Genética, Universidade de São Paulo, Ribeirão Preto, Brazil at ± 25°C on a 12:12 h LD cycle on cornmeal-yeast-agar food. All emerging adults were collected from zero to two days after eclosion from each culture bottle, separated by sex using CO_2 _anesthesia, and placed into separate vials. Flies were aged at least 10 days before use to ensure sexual maturity. In all experiments, fly age ranged from 10 to 16 days. All procedures, experiments, and CHC extractions requiring live flies were carried out in the Sene-Manfrin laboratory in Ribeirão Preto because current Brazilian regulations prohibit exporting these species. CHC quantification was performed at the University of Arkansas and CHC characterization by gas chromatography-mass spectrometry (GCMS) was carried out at Montana State University. Despite observations that rearing substrates can influence CHC profiles [17,26], it was not possible to assess CHC variation with cactus-reared flies as host plant-microbe relationships necessary to culture all of these species on fermenting cactus tissues are not well studied. As all flies were cultured under common laboratory conditions, CHC variation should reflect population, sex, and species differences.

**Table 1:** Description of the collection sites for the *D. buzzatii *species cluster stocks used in this study.

Species	Stock Number	Location (City and State)	Geographic Coordinates	Year of Collection
*D. antonietae*	J27A6M	1. Santiago - Rio Grande do Sul (RS)	29°11'S, 54°50'W	1998
	J41P1M	2. Serrana - São Paulo (SP)*	21°13'S, 47°35'W	1999
*D. borborema*	B17.2	3. Morro do Chapéu - Bahia (BA)*	11°56'S, 40°01'W	1974
*D. buzzatii*	J26A45	4. Osório - Rio Grande do Sul (RS)*	29°53'S, 50°10'W	1998
	J66M2	5. Furnas - Minas Gerais (MG)	20°37'S, 46°15'W	2000
	J92A21	6. Milagres - Bahia (BA)	12°51'S, 39°53'W	2002
	N57S27	7. Serra do Cipó - Minas Gerais (MG)	19°19'S, 43°37'W	2006
*D. gouveai*	J18M1	8. Pirenópolis - Goiás (GO)	15°51'S, 48°57'W	1997
	J67M1	9. Analândia - São Paulo (SP)	22°09'S, 47°42'W	2000
	J75L11	10. Cristalina - Goiás (GO)	16°46'S, 47°36'W	2001
	J78M1	11. Ibotirama - Bahia (BA)*	12°16'S, 43°04'W	2001
*D. koepferae*	B20D2	12. Tapia - Tucumán (TU)*	26°32'S, 65°15'W	1970
*D. serido*	J92A91M	13. Milagres - Bahia (BA)*	12°51'S, 39°53'W	2002
	N20A3	14. Arraial do Cabo - Rio de Janeiro (RJ)	22°57'S, 42°01'W	2004
	N21M1	15. Macaé - Rio de Janeiro (RJ)	22°19'S, 41°45'W	2004
	N34M3	16. Mucuri - Bahia (BA)	17°58'S, 39°29'W	2005
*D. seriema*	D40F1	17. Morro do Chapéu - Bahia (BA)*	11°56'S, 40°01'W	1990
	N57S4	18. Serra do Cipó - Minas Gerais (MG)	19°19'S, 43°37'W	2006

All 18 populations were used for CHC quantification. One population of each species, indicated by an asterisk (*), was used for CHC characterization by gas chromatography-mass spectrometry (GCMS). Thirteen out of 18 populations had data available for both *per *gene and CHCs and were used for phylogenetic reconstruction (shown as underlined). Except for *D. koepferae *from Argentina, all other populations were collected in Brazil.

### Chemical Analysis of CHCs

One population of each species (Table 1) was used to identify epicuticular hydrocarbon components in males and females. The most abundant CHCs were characterized by GCMS following Etges and Jackson [7]. In short, hundreds of adults of each species were separated by sex, allowed to mature, and then rinsed with HPLC grade hexane in Biosil™ mini-columns. Extracts were dried at 40°C under a stream of nitrogen and sealed/stored at -20°C. Each extract was analyzed with a Hewlett Packard 5890 GC fitted with a 12-m HP-1 fused silica column programmed at 150°C to 300°C at 10°C/min and held at 300°C for 5 min. The injector and detector temperature (Hewlett Packard 5971 mass selective detector) was 280°C. Extracts were redissolved in hexane containing 100 ng/fly of docosane (C_22_) as an internal standard. The unsaturated CHCs were derivatized with dimethyl disulfide (DMDS), and the resulting thiomethyl derivatives were analyzed by GCMS to identify the positions of the double bonds [24].

### CHC Variation among Populations and Species

Eighteen populations, including at least one geographical stock of each species, were used to quantify variation in male and female CHCs. Preliminary CHC classification was determined by comparing the retention times of each observed CHC component from the *D. buzzatii *cluster species with those of the *D. mojavensis *cluster [7]. In all cases, the retention times of most of the major CHCs were very similar to those of *D. mojavensis *indicating a remarkable degree of CHC conservation among these distantly related species groups. Ten aged, virgin adult flies for each sex of 18 different populations (Table 1) were individually immersed in HPLC hexane for 10 minutes with agitation, dried at 40°C, stored at -20°C, and returned to the University of Arkansas. Each extract was redissolved in 5 μl heptane containing 360 ng of docosane (C_22_) as an internal standard [26]. One μl of sample was analyzed by capillary gas-liquid chromatography in an automated Shimadzu GC-17H High Speed FID/GC fitted with an AOC-20i autosampler (Shimadzu Scientific, Columbia, MD). Injector and detector temperatures were set to 345°C with the injector port in split mode. Running temperatures started at 200°C and increased to 345°C at 10°C/min, with a hold at 345°C for 7 min [49].

### Statistical Analyses

CHC amounts were estimated by analysis of peak integrations using Class VP 4.2 software provided by Shimadzu. Each sample amount was normalized by the measured amount of docosane and all data were expressed as nanograms per fly of CHCs. We quantified amounts of 36 peaks in each sample after eliminating 18 peaks with areas that accounted for less than 1% of the total hydrocarbon abundance in at least one fly in all populations. All data were assessed for normality with PROC UNIVARIATE using SAS 9.1 [50] and log_10 _transformations improved normality. Nested multivariate analysis of variance was used to assess CHC variation among species and populations nested within species were considered random effects. The main effects in the model included species, sex and population nested within species and the interactions were species × sex and × population nested within species.

Five canonical discriminant function (CDF) analyses (PROC CANDISC) were performed to summarize CHC variation along continuous scales representing orthogonal axes of CHC covariation that best separated populations/species and to help visualize group differences. Out of the 36 peaks scored, 15 minor peaks were eliminated prior to the CDF analyses due to missing values. Consequently, a total of 21 peaks were used in the five different CDF analyses performed. First, we carried out a CDF analysis using all data, i.e. 18 populations/species (Table 1) to explore the overall magnitude of CHC differentiation in our data. This procedure was followed by a linear discriminant function analysis (PROC DISCRIM) using the same dataset to classify individuals based on species, population and sex. Second, we performed a CDF analysis without the four populations of *D. serido*, i.e. 14 populations/species, due to large, unanticipated intraspecific CHC variation in this species (see results). Third, we used CDF analysis to generate CVs for character mapping, i.e. for those populations used in the phylogenetic reconstruction (see description below). Thirteen out of 18 populations from which data was available for both *per *gene and CHCs were used in the character evolution analysis. In this third analysis, besides the 13 populations/species of the *D. buzzatii *cluster we also included the three species of the *D. mojavensis *cluster. We did not pool the sexes (as in the first and second CDF analyses) because we were interested in sex-specific CHC evolution. We performed the CDF analysis with females and males together so that male and female species-specific CDF scores could be compared on a common scale, but separated the data by sex to evaluate CHC evolution in the character reconstruction analyses. Finally, a fourth and fifth CDF analyses were also used in character mapping and were similar to the third analysis, except that in the fourth analysis we did not include the species of the *D. mojavensis *cluster and in the fifth analysis the *D. serido *populations were excluded. For all five CDF analyses, Pearson correlation coefficients were calculated between individual CHC amounts and canonical scores for each CHC for the first five CVs with PROC CORR to determine which CHC peaks were significantly associated with these canonical variates. Lastly, we conducted stepwise discriminant analyses (PROC STEPDISC) for each of the five datasets used in the CDF analyses to evaluate which CHC peaks most contributed to the variation between populations.

### Mantel Tests

We were also interested in whether geographic distance between populations distributed over such a large area (Figure 1) might explain some of the interspecific variation in CHCs due to factors like ambient ecological differences, sexual selection, or genetic drift. Our null hypothesis was that geographic distance measured in kilometers should be unrelated to overall CHC differences between populations. We performed Mantel tests using Manteller software [51] and compared female and male CHC matrices based on Euclidean distances with a geographic distance matrix of 18 populations/species. Pair-wise, great circle distances between populations were calculated using the "Haversine" formula [52].

### Phylogenetic Reconstruction

Originally, we combined chromosomal inversion differences [41,53] with the *per *gene [46] and mtDNA COI sequence data [44] to reconstruct phylogenetic relationships for the seven *D. buzzatii *cluster species. Chromosome inversions have high phylogenetic utility in *Drosophila *[42], but because only four inversions are unique and thus phylogenetically informative in the *D. buzzatii *species cluster (Figure 2), populations of the same species were all coded with the same inversions. For all species, inversions were coded as present (1) or absent (0). Although the phylogeny based on COI sequences did not recover all populations of the same species in the same clade [44], we thought the mtDNA data could still be useful in combination with chromosomal inversions and the *per *gene. However, the phylogeny produced by combining all three data sets was clearly driven by the COI sequence data (Additional File 1: Figure S1). We followed Santos et al. [54] in concluding that these mtDNA COI data did not provide clear phylogenetic relationships for these species, either alone or when combined with nuclear markers. Thus, only *per *+ inversion data were used in the phylogenetic reconstruction.

We only used populations/species from the *D. buzzatii *cluster from which *per *gene and CHC data were available (13 out of 18 populations) since the reconstructed phylogeny was used later to study CHC evolution (see below). Populations used in the *per *phylogeny [46] are indicated in Table 1. We also included two species used as outgroups by Franco et al. [46], i.e. *D. mojavensis *and *D. hydei*. Because no CHC data were available for *D. hydei *this species was removed before the tree was used for reconstruction of CHC evolution. The published *per *sequences were aligned using Mega version 4 [55]. Phylogenetic analysis of the *per *gene + chromosomal inversion data was performed using PAUP* 4.0 [56]. Maximum parsimony was used to search for optimal tree(s) and heuristic searches were carried out with 100 random addition analyses and tree bisection reconnection (TBR) branch swapping. Nodal support was obtained using bootstrap analysis (1,000 replicates).

### Mapping CHCs onto the Phylogeny

Patterns of character evolution were inferred by mapping CHC canonical variates (CVs) (See Statistical Analyses) onto the reconstructed phylogeny using Mesquite 2.6 [57]. The CVs were mapped onto the first out of six most parsimonious trees instead of the strict consensus tree because one of the models used, Squared Change Parsimony Gradual (see below), relies on branch length information. Besides *D. mojavensis*, we also added the other two species of the *D. mojavensis *cluster, *D. arizonae *and *D. navojoa*, as a sister group to the *D. buzzatii *cluster. We included the *D. mojavensis *cluster in the analysis because its phylogeny is well established [58,59], CHC data were available [7], and we were interested in its evolution as well. Because the number of species used in the phylogeny can influence the detection of phylogenetic signal [2] where higher numbers of species (17 - 20) can increase the power of the analysis, adding these species is justified and should help to avoid type II error, i.e. failure of rejecting the null hypothesis of no phylogenetic signal when in reality there was a significant relationship between CHC profiles and the phylogeny. We also performed two other character reconstruction analyses: one with just the populations/species of the *D. buzzatii *cluster and another without the populations of *D. serido*. In the former analysis we wanted to assess patterns of character evolution without the effects of outgroup species and in the latter analysis without the influence of these highly divergent populations.

Because reconstruction methods have different assumptions, they can lead to different reconstructions of ancestral states [60-62] and also influence the detection of phylogenetic signal. Therefore, we decided to employ three different parsimony methods, i.e. Linear Parsimony (LP), Squared Change Parsimony Gradual (SCPG), and Squared Change Parsimony Punctuated (SCPP) to determine whether they would yield different results. LP algorithms minimize the sum of the absolute values of changes on the branches of the tree [63]. The LP method does not use branch length information and assumes stabilizing selection as the model of evolutionary change [60]. Both SCPG and SCPP algorithms [64] minimize the sum of the squared changes on the branches of the tree. The SCPG method calculates squared changes based on branch lengths from the reconstructed tree assuming a Brownian motion model, i.e. steady gradual change (SCPG). Conversely, SCPP produces squared changes based on all branches lengths set to one with equal rates of evolution along each branch to simulate a model of punctuated evolution, where changes occur at speciation events [60,65,66].

We assessed congruence between the CHC canonical variates and the phylogeny (reference tree) by testing for the degree of phylogenetic signal revealed by these parsimony methods. Our null hypothesis was that non-phylogenetic influences such as developmental noise, ecological effects such as rearing conditions, or species-specific sexual selection have shaped CHC profiles such that CHC evolution was independent of species evolution. Our alternative hypothesis was that significant phylogenetic signal should be observed due to the phylogenetic affinities of these populations and so CHC variation should be correlated with species evolution. Evidence for phylogenetic signal in our data was evaluated in all three parsimony reconstruction algorithms by randomly modifying the reference tree, i.e. reshuffling the terminal taxa 10,000 times to generate a population of random trees for each character (female and male CVs). These trees with reshuffled taxa were then compared with the reference tree to test whether CHC distributions were more conserved than expected by chance alone. We concluded that there was phylogenetic signal if the number of parsimony character steps in the reference tree was less than in 95% of the trees with reshuffled taxa, i.e. values that fell on the extreme left of the distribution had fewer changes than expected by chance (Additional File 2: Figure S2). Alternatively, if CHC variation among closely related species was less than expected given their phylogenetic affinities, i.e., if the mean parsimony character steps for the reference tree fell on the extreme right of the reshuffled distribution, we interpreted this outcome as a result of more CHC differentiation than expected by chance alone [see [67] for details].

The detection of phylogenetic signal was also examined with the test for serial independence (TFSI), described in Abouheif [68], and available in the program Phylogenetic Independence 2.0 [69]. We decided to use TFSI as an alternative to the parsimony models because it does not assume a model of evolutionary change or require branch lengths. While this can be problematic because topology alone cannot provide all information about species similarity [2], it can be a strength if the branch lengths or model of evolutionary change are not known or accurate [68]. Furthermore, parsimony results can be misleading if the model of evolutionary change differs significantly from gradual change, i.e. when rates of evolution are rapid and/or rates of gains and losses are not equal [60,62]. For all three parsimony methods and TFSI, *p*-values were corrected for multiple comparisons via false discovery rate (FDR) analysis [70,71].

## Results

### Chemical Composition of CHCs

All seven species and both sexes in the *D. buzzatii *cluster had CHCs with carbon chain lengths ranging from C_29 _to C_39 _(Additional File 3: Figure S3). The CHCs of adult flies were composed of three structural classes: mono-methyl-branched alkanes, *n*-alkenes, and alkadienes (Table 2). Mono-methyl-branched alkanes comprised both the C_28.65 _and C_30.65 _peaks. The former had the same composition for all species and both sexes (i.e. 2-methyloctacosane), while the latter varied among species, composed of either a mono-methyl-branched alkane (2-methyltriacontane) or *n*-alkenes [(*Z*)-5-hentriacontene or (*Z*)-9-hentriacontene]. In fact, C_30.65 _was one of the four peaks showing qualitative differences between sexes, i.e., C_30.65 _was composed of 2-methyltriacontane in *D. gouveai *♂, *D. seriema *♀ and *D. koepferae *♀, but composed of (*Z*)-9-hentriacontene in *D. gouveai *♀, *D. seriema *♂ and *D. koepferae *♂. Peaks C_37 _and C_36.5 _were also sexually dimorphic, but results were only available for *D. gouveai *(Table 2).

**Table 2:** Key mass spectra peaks used in the identification of CHCs from the *D. buzzatii *species cluster.

			Diagnostic ions *(m/z)*		
				
Carbon Number	ECL^a^	Hydrocarbon^b^	Untreated	Dimethyl Disulfide Derivative	Notes^c^
29	28.65	2-methyloctacosane	365, 393, 408		All species and sex
31	30.65	2-methyltriacontane	393, 421, 436		*D. serido *♀; *D. gouveai *♂; *D. seriema *♀; *D. koepferae *♀; *D. antonietae *♀and ♂
		(*Z*)-5-hentriacontene	434	117, 411	*D. serido *♂
		(*Z*)-9-hentriacontene	434	173, 355	*D. gouveai *♀; *D. seriema *♂; *D. koepferae *♂; *D. buzzatii *♀
33	33 br3	(*Z*)-14-; (*Z*)-12-; and (*Z*)-10-tritriacontene	462	187, 215, 243, 313, 341, 369	*D. gouveai *♀ and ♂; *D*. *seriema *♀ and ♂; *D. koepferae *;*D*. ♀ *buzzatii *♀ and ♂
		(*Z*)-16-; (*Z*)-15-tritriacontene	462		*D. koepferae *♂
	32.47	(*Z*)-8-tritriacontene	462	159, 397	All species and sex, except *D. antonietae *♀ and ♂
	32.56	(*Z*)-6-tritriacontene	462	131, 425	All species and sex, except *D. serido *♀ *D. antonietae *♀ and ♂
	32.79	(*Z*, *Z*)-5,25-tritriacontadiene	460	117, 437	*D. serido *♀ and ♂
		(*Z*, *Z*)-7,25-tritriacontadiene	460	145, 409	*D. gouveai *♀ and ♂; *D. seriema *♀ and ♂; *D. koepferae *♀
	32.86	(*Z*, *Z*)-7,25-tritriacontadiene	460	131, 423	*D. serido *♀ and ♂
34	34 ene	(*Z*)-16-; (*Z*)-14-tetratriacontene	474	215, 243, 271, 299, 327, 355	*D. gouveai *♀ and ♂; *D. koepferae *♀ and ♂; *D. buzzatii *♀ and ♂
35	35 ene 1	(*Z*)-16-; (*Z*)-14-; (*Z*)-12-pentatriacontene	490	215, 243, 271, 313, 341, 369	*D. gouveai *♀ and ♂; *D. seriema *♀ and ♂
		(*Z*)-16-; (*Z*)-14-pentatriacontene	490	243, 271, 313,	*D. koepferae *♀ and ♂
		(*Z*)-14-; (*Z*)-12-pentatriacontene	490	215, 243, 341, 369	*D. buzzatii *♀
	35 ene 2	(*Z*)-10-pentatriacontene	490	187, 397	All species except *D. serido *♀ and *D. antonietae *♀ and ♂
	35 ene 3	(*Z*)-8-pentatriacontene	490	159, 425	All species except *D. serido *♀ and *D. antonietae *♀ and ♂
	34.66	(*Z*, *Z*)-5,25-pentatriacontadiene	488	187, 395	*D. serido *♀ and ♂
		(*Z*, *Z*)-9,25-pentatriacontadiene or (*Z*, *Z*)-9,27-pentatriacontadiene	488	173, 423	*D. gouveai *♀ and ♂
		(*Z*, *Z*)-8,26-pentatriacontadiene	488	159, 409	*D. seriema *♀ and ♂
	34.79	(*Z*, *Z*)-7,27-pentatriacontadiene	488	145, 437	*D. serido *♀ and ♂
		(*Z*, *Z*)-7,27-pentatriacontadiene or (*Z*, *Z*)-7,25-pentatriacontadiene	488	145, 437	*D. gouveai *♀ and ♂
		(*Z*, *Z*)-6,28-pentatriacontadiene		131, 453	*D. seriema *♂
37	37	(*Z*)-16-; (*Z*)-18-; (*Z*)-14-heptatriacontene	517	243, 271, 299, 313, 341, 369	*D. gouveai *♂
		(*Z*, *Z*)-10,23-Heptatriacontadiene	517	187, 423	*D. gouveai *♀
	36.5	(*Z*)-16-; (*Z*)-18-; (*Z*)- 14-heptatriacontene	517	243, 271, 299, 313, 341, 369	*D. gouveai *♂
		(*Z*, *Z*)-9,27-heptatriacontadiene	517	173, 437	*D. gouveai *♀
	36.7	(*Z*)-16-; (*Z*)-18-; (*Z*)-14-heptatriacontene	517	243, 271, 299, 313, 341, 369	*D. gouveai *♀ and *D. gouveai *♂

Only major peaks were scored. See Table 1 for description of the populations used.

*^a ^*Equivalent chain length calculated as in Stennett and Etges [26].

^b ^Isomer order ranges from major to minor abundance.

^c ^Species/sexes that are not included had hydrocarbons that could not be identified.

All other observed peaks were composed of either monoenes or dienes. Several peaks were comprised of mixtures of positional monoene isomers (Table 2) where the location of double bonds was mainly at even-numbered carbons (e.g. (*Z*)-8-tritriacontene and (*Z*)-10-pentatriacontene). Alkadienes were also present in more than one positional isomer, but the double bonds were located mostly at odd-numbered carbons (e.g. (*Z*, *Z*)-7,25-tritriacontadiene and (*Z*, *Z*)-5,25-pentatriacontadiene). The composition of some peaks was not determined because these samples proved difficult to derivatize with DMDS.

### Quantitative Variation in CHC Profiles

Quantitative variation in CHCs was much more prominent than chemical differences between species. CHC variation due to sex, species, and population and all interactions were significant (Table 3). Out of the 36 peaks analyzed, 12 major peaks accounted for ca 85% of the total hydrocarbons for all 18 populations/sexes analyzed (Additional File 4: Table S1). Mean total hydrocarbon amount per fly was 878.68 ng ± 21.71 ( ± 1 SE). All pair-wise squared Mahalanobis distances between species were significant (*P *< 0.0001), as were differences among populations (Wilks λ = 0.0000, *F *= 57.11, *P *< 0.0001). MANOVA revealed significant species and population specific sexual dimorphism revealed by a species × sex interaction term (30/36 peaks) and sex × population nested within species term (27/36 peaks) (results not shown). Thus, sexual dimorphism in CHC profiles was a significant source of variation in populations of all seven species. Linear discriminant function analysis based on 18 populations/species correctly assigned 98.2% and 96.6% of individuals (out of 298 total) to their correspondent populations and species, respectively. The classification based on sex correctly assigned 76% and 80% of females and males, respectively. Therefore, CHC variation in the *D. buzzatii *cluster was largely species-, population-, and sex-specific.

**Table 3:** Nested MANOVA results for 36 CHC peaks in 18 populations/species of *D. buzzatii *cluster species.

Source of Variation	Wilks λ	*F *value	df	*P*
Species	0.0000	130.35	216, 1099.6	<0.0001
Sex	0.1944	21.18	36, 184	<0.0001
Population(Species)	0.0000	21.91	396, 1973.9	<0.0001
Sex × Population(Species)	0.0018	4.09	396, 1973.9	<0.0001
Species × Sex	0.0017	9.87	216, 1099.6	<0.0001

Population effects were nested within species.

**Figure 3:**
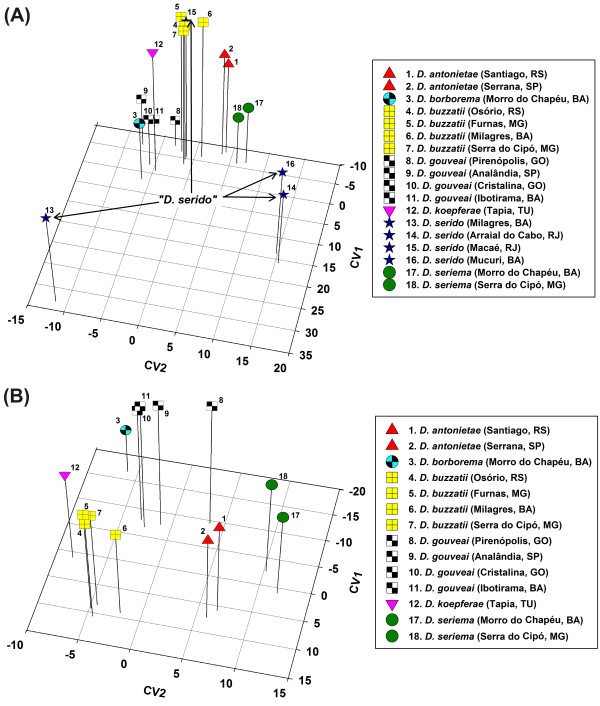
**A, B. Three dimensional plots of the D. buzzatii species cluster based on the first three canonical variables (CVs) obtained from 21 CHC components analyzed.A)** Plot of the 18 populations/species.Altogether, the first three CVs explained 83% of the variance in the data (CV1 = 48%, CV2 = 20%, and CV3 = 15%) See Additional File 5: Table S2 for details. All Mahalanobis distances between populations were significant (*P *< 0.0001). Arrows denote the highly divergent *D. serido *populations. Numbers represent the localities of the eighteen populations used in the CHC analysis (see Table 1 and Figure 1). **B) **Plot of the 14 populations/species of the *D. buzzatii *cluster after deleting the four *D. serido *populations. Altogether, the first three CVs explained 85% of the variance in the data (CV1 = 46%, CV2 = 27%, and CV3 = 12%). See Additional File 6: Table S3 for details.

The first five canonical variates from the CDF analysis for all 18 populations/species accounted for 94% of the total hydrocarbon variation (Additional File 5: Table S2) and grouped populations of the same species together except for *D. serido *populations that were highly divergent and showed large discordance with the other populations/species (Figure 3A). The first canonical variate (CV1) was largely influenced by the divergence among *D. serido *populations. All four populations of *D. serido *differed significantly in CHC composition, and three of these populations were more different from each other than most of the other species (Figure 3A). *D. serido *populations from Mucuri, Bahia and Arraial do Cabo, Rio de Janeiro (Figure 1) clustered together but were isolated from all other populations/species. The Milagres, Bahia population of *D. serido *was the most divergent population in the analysis and was completely isolated from all other populations/species including the other *D. serido *populations. The fourth population of *D. serido*, from Macaé, Rio de Janeiro, had CHC profiles similar to those of *D. buzzatti *populations (Figure 3A). Therefore, we suspected that the Macaé population was contaminated with *D. buzzatii *in lab culture because this population was collected less than 100 km away from Arraial do Cabo (populations 14 and 15, Figure 1, Table 1), and it is unlikely that these two populations would be so different in CHC profiles. The second canonical variate (CV2) separated populations of the same species, but was still dominated by the striking differences caused by the *D. serido *populations. Since *D. serido *was apparently causing so much of the overall CHC variation and obscuring the differences between the other species, we performed a second CDF analysis excluding all four *D. serido *populations (Additional File 6: Table S3). We observed not only reduced total variation in this analysis (see axis range in Figure 3B) but also a clearer separation of the species (CV1) and populations of the same species (CV2). Overall, the striking degree of intraspecific CHC variation found in *D. serido *populations suggests the presence of at least two cryptic "*D. serido*" lineages.

**Figure 4:**
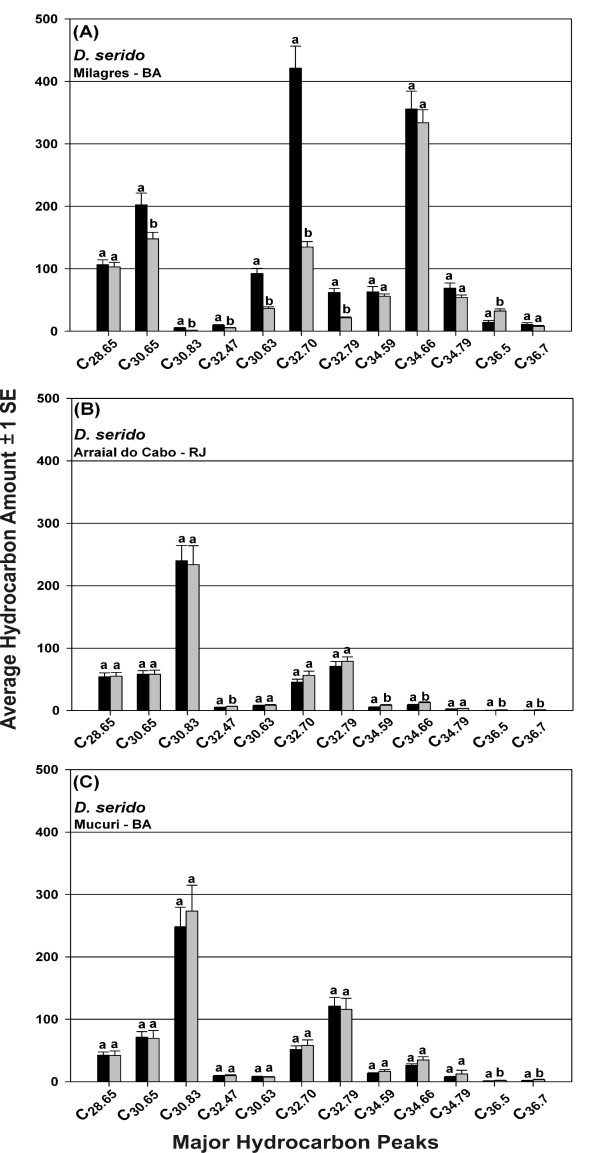
**A - C. Epicuticular hydrocarbon amounts (average ± 1 SE) for 12 major hydrocarbon peaks of females (black) and males (gray) of 3 populations of *D. serido *(*D. serido *from Macaé is not shown)**. For each peak same letters represent non-significant means between females and males. Components are referred to by their equivalent chain lengths.

We also attempted to identify which CHCs were responsible for these striking population/species differences. First, we used stepwise discriminant function analysis with both forward and reverse variable entry to identify which of the 21 CHC peaks was/were driving the differences among populations. All but one of the 21 CHC components were significantly correlated with the discriminant function, *P *< 0.0001, with partial correlations of 0.149 to 0.905 (Additional File 7: Table S4). Next, we sequentially deleted individual peaks and performed additional CDF analyses in an attempt to identify which CHCs caused the large differences due to *D. serido *(Figure 3A). We started by removing the C_30.83 _component, a CHC with one of the largest partial correlations with the discriminant function (Additional File 7: Table S4) that was found in significantly higher amounts in *D. serido *populations from Mucuri and Arraial do Cabo (Figure 4), but was absent or in negligible amounts in all other populations/species (Additional File 4: Table S1). Removal of the C_30.83 _peak did not eliminate the large *D. serido *population differences so we deleted another large component, C_32.47_, which resulted in eliminating most of the CHC differences that separated *D. serido *from Milagres, Bahia from the other species (results not shown). Thus, there were population-specific CHCs that seemed to be driving these extraordinary intraspecific differences in CHCs, but the general pattern of CHC differentiation between these populations involved quantitative variation in most of the CHCs scored. Results of the Mantel tests [51] assessing associations between CHC differentiation among populations/species and geographic distance were not significant (females: r = - 0.049, *P *= 0.596; males: r = - 0.078, *P *= 0.784).

### Phylogenetic Reconstruction and CHC Character Mapping

**Figure 5:**
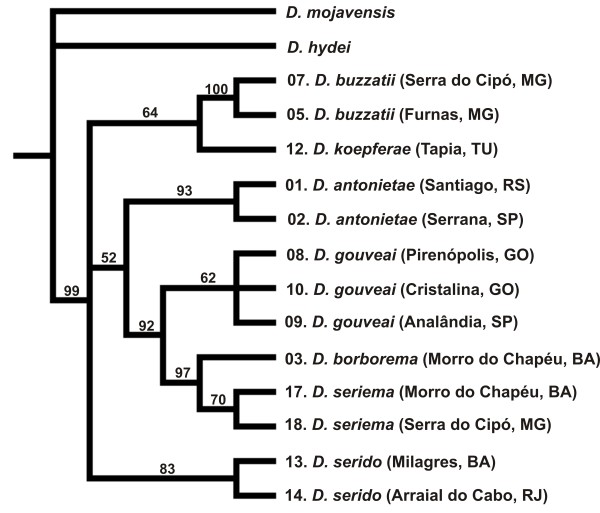
**Strict consensus tree of six most parsimonious trees (Length = 166, CI = 0.82; RI = 0.76) of the populations/species of the *D. buzzatii *cluster plus two outgroup species (*D. mojavensis *and *D. hydei*) inferred from chromosomal inversions **[41]**and *****period *****gene data **[44]. Bootstrap support (1,000 replicates and 100 random additions) is shown above the branches. Only bootstrap values above 50% are shown. The numbers before the species names represent the localities where the populations used for CHC analysis were collected. Only populations that had data for both CHC and *per *gene were used to reconstruct the phylogeny, i.e. 13 out of 18 populations (see Table  1).

The parsimony analysis resulted in six equally most parsimonious *per *gene + chromosome inversion trees (Additional File 8: Figure S4) of 166 steps (CI = 0.82; RI = 0.76). The strict consensus tree (Figure 5) produced a well-resolved phylogeny that clustered all populations/species of the *D. buzzatii *cluster together (bootstrap value 99%). We decided to use the first out of six most parsimonious trees for character mapping because this tree was the one that closest resembled the strict consensus tree. In fact, all six parsimony trees had very similar topologies (Additional File 8: Figure S4). Tree 1 and tree 2 had the same topology except that tree 2 had a polytomy in the clade containing populations of *D. gouveai*. Since polytomies had to be resolved prior to performing the character reconstruction analyses, these two trees were equivalent for character mapping. Trees 3 and 4 also had the same order of taxa observed for trees 1 and 2 and differed from them only in the arrangement of internal branches. Trees 5 and 6 differed from the first four trees by clustering *D. serido *populations in the same clade containing the *D. buzzatii *populations and *D. koepferae *rather than in a separate clade.

**Figure 6:**
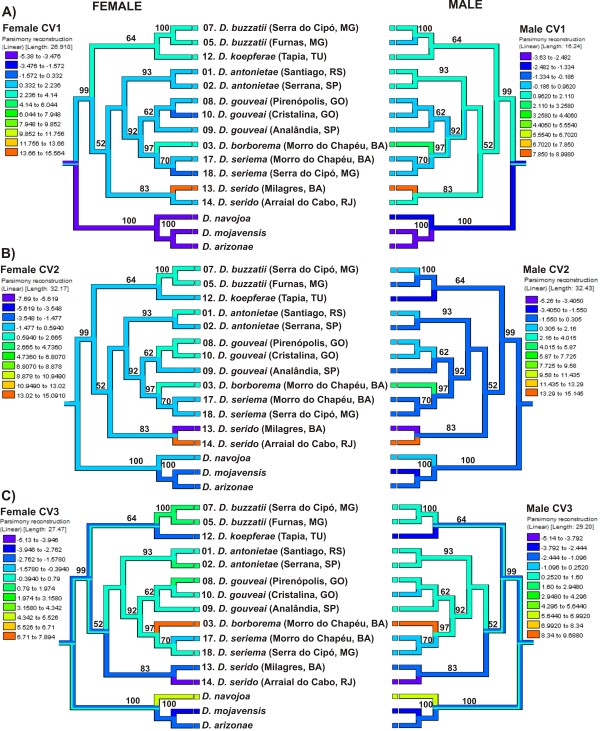
**A - C. Phylogenetic character mapping using the linear parsimony model with the first three canonical variates (CV1-CV3) based on female and male CHCs**. Both sexes were analyzed together in the same CDF analysis to avoid scale effects but female and male canonical variates (CVs) were mapped separately onto the reconstructed phylogeny (see left and right trees). This phylogeny represents a most parsimonious tree (one of six trees) of the populations/species of the *D. buzzatii *cluster inferred from chromosomal inversions [41] and the *period *gene [49]. One of the outgroup taxa, *D. hydei*, was removed prior to the character state reconstruction because no CHC data was available for this species. The other two species of the *D. mojavensis *cluster, *D. arizonae *and *D. navojoa*, were added to the analysis. Bootstrap values (shown above the nodes) were based on 1,000 replicates and 100 random additions. Only bootstrap values above 50% are shown. Bootstrap support for species of  *D. mojavensis *cluster was based on Durando et al. [58].

In the reconstructed phylogeny based on the populations/species of the *D. buzzatii *cluster plus the *D. mojavensis *cluster, significant phylogenetic signal was observed for female CV1 (Table 4) when linear parsimony (LP) (Additional File 2: Figure S2), squared change parsimony punctuated (SCPP) and the test of serial independence (TFSI) were used, but no correlation was observed when squared change parsimony gradual (SCPG) was used. For male CV1, all four methods yielded significant phylogenetic signal (Table 4). Female CV2 displayed significant phylogenetic signal only with TFSI and female and male CV5 were significant only with LP method. For CV2, CV3, and CV4, no concordance between CHC profiles and the phylogeny was detected with any of the three parsimony methods. Similar results were obtained with TFSI, except for female CV2, which was positive for phylogenetic signal (Table 4). Figure 6A, B and 6C shows the first three female and male CVs mapped onto the phylogeny using the LP method. For CV1, the CHCs of the *D. mojavensis *cluster clearly differed from those of the *D. buzzatii *cluster (Figure 6A). Within the *D. buzzatii *cluster, female *D. buzzatii *and *D. koepferae *had very similar CHC profiles that were not shared among the other species, except for *D. borborema*. For males, *D. buzzatii *and *D. koepferae *also had similar profiles, but this similarity was also shared with other taxa, mainly with *D. antonietae *populations and *D. serido *from Arraial do Cabo. The most differentiated population in terms of CHCs was *D. serido *from Milagres, Bahia (Figure 3A and 4). No phylogenetic signal was observed for CV2 and CV3 as indicated by the somewhat random distributions of CHC profiles across the tree (Figure 6B, C). For CV2, another *D. serido *population from Arraial do Cabo, Rio de Janeiro had the most divergent CHC profile (Figure 6B) whereas for CV3, *D. borborema *from Morro do Chapéu, Bahia was the most divergent group (Figure 6C). The influence of sex on CHC variation was diminished because we included male and female CHC data for each population/species to generate common canonical variables all on the same CDF scales.

**Table 4:** Analysis of congruence between the chromosomal inversion plus *per *gene phylogeny and CHC data.

PARSIMONY METHODS										TEST FOR SERIAL INDEPENDENCY (TFSI)	
			
	Linear Parsimony (LP)			Squared Change Parsimony Gradual (SCPG)			Squared Change Parsimony Punctuated (SCPP)				
				
*Characters*	*Reference Tree*	*Random Trees*	*P*	*Reference Tree*	*Random Trees*	*P*	*Reference Tree*	*Random Trees*	*P*	Observed Mean C-Statistics	*P*
Female CV1	26.91	37.14	**0.0012**	37.17	78.63	0.0921	105.88	166.56	**0.0026**	0.3615	**0.0020**
Female CV2	32.17	32.75	0.3317	94.71	75.49	0.7182	228.03	158.34	0.9553	-0.3217	**0.0090**
Female CV3	27.47	30.34	0.1287	26.10	39.20	0.1756	69.06	82.77	0.1976	0.1094	0.2460
Female CV4	26.61	29.81	0.0931	31.77	42.13	0.3025	66.29	89.39	0.0683	0.1593	0.2210
Female CV5	26.31	37.30	**0.0040**	23.43	51.03	0.0306	59.80	108.28	0.0223	0.2981	0.0480
Male CV1	16.24	23.37	**0.0004**	11.88	28.88	**0.0002**	32.43	61.08	**0.0004**	0.447	**0.0010**
Male CV2	32.43	32.13	0.4783	83.85	69.03	0.6803	197.64	146.13	0.9652	-0.2548	0.0490
Male CV3	29.20	30.75	0.2032	35.84	47.76	0.2850	91.14	101.18	0.2689	0.0579	0.2930
Male CV4	28.92	31.11	0.1142	53.11	56.40	0.4929	99.37	119.68	0.1340	0.0761	0.3000
Male CV5	27.39	36.75	**0.0080**	43.49	51.24	0.8830	75.87	108.81	0.0699	0.1669	0.1590

The reconstructed phylogeny used in the character evolution analysis represents the first out of six most parsimonious trees and was based on 13 populations/species of the *D. buzzatii *cluster (see Table 1) plus three species of the *D. mojavensis *cluster. CDF analysis was based on 21 CHC peaks to generate the canonical variates (CVs). Three different parsimony methods were used in Mesquite [57]: linear parsimony (LP), squared-change parsimony assuming a gradual model of evolution (SCPG), and squared-change parsimony with a punctuated model of evolution (SCPP). In all three models, presence of phylogenetic signal for each character (i.e. female and male CVs) was assessed by comparing the mean parsimony character steps from the reference tree (as shown on Figure 6) with those of a population of random trees. Terminal taxa were reshuffled 10,000 times to generate the random trees. Phylogenetic signal was positive when the mean parsimony character steps for the reference tree were significantly smaller than the mean parsimony character steps for the random trees. See Additional File 2: Figure S2 for details. The detection of phylogenetic signal was also examined with the test for serial independence (TFSI) run with 1,000 replicates using the program Phylogenetic Independence 2.0 [69]. *P*-values in bold represent significant values after false discovery rate (FDR) analysis. See Additional File 12: Table S8 for FDR calculations.

In order to dissect these patterns of covariation further, we evaluated the loadings of each CHC on the canonical variates. Almost all individual CHCs were significantly correlated with each of the first five CVs (Table 5). For CV1, all CHC peaks significantly contributed to the variation, except for C_32.56_, but three CHCs with the highest loadings, C_33br3_, C_32.47_, and C_35ene3 _and to a lesser extent two C_35 _components, C_35ene3 _and C_34.66_, best discriminated among these populations. Only two of these components, C_32.47 _and C_34.66_, represented significant proportions of total CHCs or were "major peaks" in these species suggesting that even relatively "minor" CHC components were responsible for these species differences that resulted in the detection of phylogenetic signal in CHC differentiation and evolution. Similar results were observed for CV2, CV3, CV4, and CV5, where most of the CHC components were significantly correlated with the CVs. When sex was considered as a variable in the model, the percentage of the variance explained by the first five CVs increased from 72% to 81% (results not shown), with the highest difference found in CV1, from 17% to 22%.

**Table 5:** The first five canonical variates based on the total canonical structure of 13 populations/species of the *D. buzzatii *cluster plus the three species of the *D. mojavensis *cluster.

Carbon Number	CHC Peak^+^	CV1 (17%)	CV2 (17%)	CV3 (14%)	CV4 (13%)	CV5 (11%)
29	C_28.65_	-0.181***	-0.381****	-0.573****	0.192***	0.006 ns
31	C_30.65_	-0.379****	-0.453****	0.102 ns	-0.356****	0.349****
	C_30.78_	-0.291****	0.100 ns	-0.199***	-0.180***	-0.422****
	C_30.83_	0.153**	0.811****	-0.361****	-0.226****	0.127*
33	C_33br2_	-0.607****	-0.283****	-0.071 ns	-0.028 ns	0.358****
	C_33br3_	0.871****	-0.341****	-0.142**	-0.140**	0.087 ns
	C_32.47_	-0.675****	-0.332****	-0.198***	-0.053 ns	0.050 ns
	C_32.56_	-0.042 ns	-0.041 ns	0.677****	-0.663****	0.093 ns
	C_32.63_	-0.146**	-0.333****	-0.425****	0.131*	-0.065 ns
	C_32.70_	0.323****	-0.382****	-0.205****	-0.264****	-0.374****
	C_32.79_	0.125*	0.111*	-0.075 ns	-0.130*	-0.665****
	C_32.86_	0.281****	0.820****	-0.307****	-0.176***	-0.062 ns
35	C_35ene1_	-0.375****	-0.220****	0.189***	-0.206****	0.405****
	C_35ene2_	-0.520****	-0.203****	0.235****	-0.115*	0.360****
	C_35ene3_	0.661****	-0.002 ns	0.196***	0.359****	-0.215****
	C_34.59_	-0.484****	-0.271****	-0.291****	0.088 ns	0.213****
	C_34.66_	-0.557****	-0.366****	-0.017 ns	-0.231****	0.359****
	C_34.79_	0.504****	0.001 ns	0.158**	0.224****	-0.355****
37	C_37_	-0.421****	-0.234****	-0.236****	0.151**	0.221****
	C_36.5_	-0.240****	-0.042 ns	0.423****	0.409****	0.485****
	C_36.7_	0.210****	0.152**	0.443****	0.705****	0.229****

Both sexes were run together in the same CDF analysis to avoid scale effects but mapped separated onto the reconstructed phylogeny (see Figure 6). Values in parenthesis represent the percentage of variance explained by each CV. Statistical significance of Pearson correlation coefficients between the original variables and canonical discriminant function loadings are indicated.

**^+ ^**Equivalent chainlengths of each hydrocarbon as defined in Table 2.

ns = not significant. * *P *≤ 0.05, ** *P *≤ 0.01, *** *P *≤ 0.01, ****P ≤ 0.0001.

Because presence of phylogenetic signal, especially for CV1, seemed to be related to CHC differences between both clusters, we performed an analysis without the *D. mojavensis *cluster in order to test whether phylogenetic signal would be present in the *D. buzzatii *cluster only. In the absence of *D. mojavensis *cluster, CV1 did not display positive phylogenetic signal (Additional File 9: Table S5). However, positive phylogenetic signal was detected for female CV3 and/or CV4 and male CV4 and CV5 (Additional File 9: Table S5) illustrating that positive phylogenetic signal for different covarying groups of CHCs was present in the *D. buzzatii *cluster even in the absence of an outgroup.

Because *D. serido *populations exhibited such high within-species CHC divergence (Figure 3A, B), we also considered the possibility that *D. serido *CHCs may have influenced the character mapping results. To test this hypothesis, we repeated the CDF analysis (Additional File 10: Table S6) and reconstructed the phylogeny without the two *D. serido *populations. In the absence of *D. serido*, male and female CV1 displayed positive phylogenetic signal with all four methods. However, as mentioned above, presence of phylogenetic signal for CV1 was influenced by including the *D. mojavensis *cluster. More strikingly was the fact that without the *D. serido *populations, all three parsimony methods (except for male SCPG) and TFSI had three or four CVs that tested positive for phylogenetic signal (Additional File 11: Table S7). Thus, the exclusion of the two rather discordant *D. serido *populations had a huge influence on our ability to detect phylogenetic signal in the differentiation of *D. buzzatii *cluster CHCs.

## Discussion

Comparative analysis of quantitative variation in CHC profiles of the *D. buzzatii *species cluster revealed that CHC evolution has been somewhat conserved and associated with the evolutionary divergence of these species. Thus, CHC differentiation among these populations has not evolved so quickly as to erase evidence of phylogenetic affinity suggesting that variation in CHCs in this group of *Drosophila *can be predicted, to some extent, by species ancestry. Here, a key observation was the degree of CHC chemical conservation between the *D. buzzatii *and *D. mojavensis *clusters (Table 2) where most molecular structures, retention times, and carbon chainlengths were conserved, but species-specific CHC amounts varied quantitatively. The *D. mojavensis *cluster is also part of the *mulleri *complex, but is endemic to North America [59,72,73]. As these species groups are restricted to different continents and diverged ca 10-15 mya [74,75], CHC biosynthesis and expression have been conserved over a large portion of the *D. repleta *group phylogeny. The most conserved chemical compounds were 2-methyloctacosane (2-MeC_28_) and 2-methyltriacontane (2-MeC_30_). These two compounds are not only shared within and between both clusters but are also found in a variety of other insect species [76]. In retrospect, such conserved CHCs may not be surprising, but few attempts have been made to assess broad-scale variation in CHCs in groups of related species. Thus, CHC evolution in these *D. repleta *group species has a significant phylogenetic component based on a core group of C_29_, C_31_, C_33_, C_35_, C_37 _and C_39 _hydrocarbons (Additional File 3: Figure S3) with additional species and population-specific variations on this theme.

The multiple functional roles for insect cuticular hydrocarbons has been appreciated for some time [77]. In arthropods with longer chain length CHCs (>20 carbon atoms), effects of desiccation are reduced because longer CHCs have higher melting temperatures [78,79], consistent with observations that xeric adapted *Drosophila *species exhibit longer chain length CHCs than mesic species [80]. Although saturated compounds, *n*-alkanes, provide increased protection against desiccation, branched and unsaturated compounds decrease melting temperatures and can cause increased rates of water loss across insect epicuticles [78]. In *Drosophila*, alkenes and alkadienes have pheromonal activity in a number of species [14,81-84]. In experimental populations of *D. melanogaster *that responded to increased desiccation conditions, CHC differences did evolve, but there were no associated changes in sexual isolation suggesting that CHCs involved in desiccation resistance were different from those used for mate choice [85]. In other insects like paper wasps [86] and honeybees [87], branched alkanes and/or alkenes are more easily identified by other individuals than linear alkanes and therefore serve as recognition cues while *n*-alkanes function primarily to reduce water loss. Given the conservation of CHC compounds in the desert-adapted *D. buzzatii *and *D. mojavensis *species groups, significant sexual dimorphism in CHC profiles (Table 3), and the presence of branched and unsaturated molecules in the CHCs of all of these species, we expect that *D. buzzatii *cluster CHCs serve as both physiological mechanisms to control transcuticular water flux as well as in chemical communication, i.e. mate recognition. Nevertheless, the role of CHCs as pheromones has yet to be confirmed in the *D. buzzatii *cluster. Preliminary results revealed undetectable pheromonal activity in CHC perfuming experiments with *D. seriema *and *D. buzzatii *even though significant amounts of CHCs were transferred between males (Oliveira et. al., unpubl. data). However, we initially chose these species for perfuming studies because of the ability to detect CHC transfers. This result may not be representative of other, more closely related species in the cluster because *D. seriema *and *D. buzzatii *were so reproductively divergent (in mate choice trials, Oliveira et. al., unpubl. data) that alterations in CHCs had little effect despite the significant CHC differences between them. Further perfuming trials with all *D. buzzatii *cluster species are clearly needed.

The detection of positive phylogenetic signal using the three different data sets: (1) *D. buzzatii *+ *D. mojavensis *cluster; (2) *D. buzzatii *cluster; and (3) *D. buzzatii *cluster (without *D. serido *populations) + *D. mojavensis *cluster (Table 4, Additional Files 9 and 11, respectively) supports the hypothesis that phylogenetic signal was strong enough to be detected by different methods independent of their assumptions. Moreover, positive phylogenetic signal was observed when just the *D. buzzatii *cluster species were used supporting that some CHCs were conserved in the cluster. These results were even more robust when the divergent *D. serido *populations were removed from the analysis. We hypothesize that CVs that were weakly correlated with the phylogeny, mainly CV2, were influenced by CHCs that may be responding to the ambient environment or other forces, i.e. these are traits involved in mate recognition like courtship songs, pheromones, or coloration that should evolve more rapidly due to sexual or stabilizing selection [88-91].

Contrasting results have been reported regarding the presence of phylogenetic signal in studies of character evolution that have implicated CHCs and other volatile compounds in mate and/or species recognition. For example, Jallon and David [13] concluded that "Hydrocarbon variations do not match the phylogeny" in eight species of the *D. melanogaster *group. Symonds and Elgar [92] reported little association between aggregation pheromone composition and phylogenetic relationships in bark beetles since closely related species were as different, if not more so, than more distantly related species. Conversely, Symonds and Wertheim [93] found that more closely related *Drosophila *species had more chemically similar aggregation pheromones and concluded that there was a positive relationship between phylogenetic distance and pheromone differentiation. Cuticular hydrocarbons in pine engraver beetles have been used to identify different species and thus have systematic value [94]. Some phylogenetic trends in species-specific CHCs were also reported in Hawaiian swordtail crickets [11]. However, known phylogenetic relationships among 78 ant species in five subfamilies showed "no similarity" to cuticular hydrocarbon differences based on chemical structures [95]. Male courtship songs were homoplasic in the *Drosophila willistoni *species complex [88], showed evidence of diversification, character loss, and reversal in the *D. repleta *group [33], and converged in green lacewings [96]. In birds, sexually selected traits like male plumage and bower characters exhibited low phylogenetic signal [97,98], while male songs were more conserved [99]. We suggest that phylogenetic diversification of insect CHCs may be more conservative than courtship songs or avian plumage characteristics because the complex underlying biochemical and physiological machinery required to synthesize and express CHCs in arthropods [9,100,101] may be more conserved than in other traits. Thus, similarity in cuticular hydrocarbon profiles among species may represent a phylogenetic constraint due to their mode of production. Certainly, more comparative studies involving mating signals will be necessary to determine whether the presence of phylogenetic signal is a rule or an exception for pheromonal or behavioral traits.

### Evolution of the *D. buzzatii *cluster and CHCs

Attempts to resolve a phylogeny using the mtDNA data [44] failed to resolve all species into individual evolutionary lineages. Specifically, *D. gouveai*, *D. serido*, and *D. seriema *show substantial geographic variation and considerable phylogenetic incongruence (Additional File 1: Figure S1). Incomplete lineage sorting or hybridization could be responsible, as well as natural selection on mtDNA function [102]. Phylogenetic reconstruction based on the nuclear *period *(*per) *gene by Franco et al. [46] resolved the relationships among *D. gouveai*, *D. borborema *and *D. seriema *(Figure 5). Although *per *grouped populations of *D. serido *together, they were not placed as a sister taxa of *D. antonietae*, as predicted by chromosomal inversion data (Figure 2). Therefore, the position of "*D. serido*" has yet to be resolved.

The large and very significant intraspecific differences in *D. serido *CHCs (Figure 3A) does not suggest a gradual model of CHC evolution, but were consistent with previously described differentiation between populations that inhabit northeastern Brazil in the caatinga (e.g. Milagres, Bahia) and those from the east coast of Brazil (e.g. Mucuri, Bahia and Arraial do Cabo, Rio de Janeiro, see Figure 1). The observation that the CHCs of the coastal *D. serido *population from Macaé, Rio de Janeiro did not match this pattern of differentiation further suggests that this stock was contaminated (see results for details). Here, the scale of intraspecific CHC variation was greater than interspecific variation for the remaining six species, and included multiple CHC components (Figure 4). Genetic divergence between populations of *D. serido *in these regions includes mtDNA haplotype differentiation [44], cytological differences, amounts of heterochromatin in metaphase chromosomes [103], and frequency differences of polymorphic inversions [41,104]. These observations together with our results showing large intraspecific CHC differences strongly suggest the presence of several more cryptic species in this group.

## Conclusions

The evolution of phenotypes and how they are shaped by phylogenetic history is a long-standing issue [1]. Our comparative approach revealed that CHC compounds were highly conserved among species. Quantitative differences in CHC profiles were more prominent yet CHCs were species-, population-, and sex-specific. The evolution of CHCs was not homogeneous as some peaks were more conserved and retained phylogenetic signal while others seemed to be evolving faster. Comparative approaches to understanding phenotypes such as CHCs with multiple functions and courtship songs in *Drosophila *have provided some insight into the patterns of trait evolution for phenotypes likely associated with mating success and reproductive isolation, as well as the challenges of xeric environments caused by desiccation and cuticular water loss. For understanding of CHC evolution, future analyses of multiple phenotypes in such groups will be necessary to evaluate whether CHC components influence water balance and/or have pheromonal activity and to determine how the type and quantity of these compounds evolve during the diversification of populations and species.

## Authors' contributions

CCO designed and conducted the experiments and performed statistical analyses. WJE assisted with the design and coordination of the study. LLJ carried out and analyzed the gas chromatography-mass spectrometry data. MHM and FMS helped with fly collection and coordination of the study. CCO and WJE wrote the manuscript. All authors read and approved the final manuscript.

## Supplementary Material

Additional file 1 **Figure S1. Strict consensus trees of the *D. buzzatii *cluster**. **A) **Phylogeny inferred from mtDNA COI data. **B) **Phylogeny based on chromosomal inversions + mtDNA + *period *gene. Bootstrap support (1,000 replicates and 100 random additions) is shown above the branches. Only bootstrap values above 50% are shown. See Figure 5 for strict consensus tree inferred from chromosomal inversions + *period *gene. Click here for file

Additional file 2 **Figure S2. Bar graphs of random distributions generated by the shuffle option in Mesquite using the Linear Parsimony Method**. **(A) **Data showing presence of phylogenetic signal. The number of parsimony character steps for the reference tree (see Figure 6A) was significantly smaller, i.e. fell on the left side of the distribution, than the number of parsimony character steps for the trees with reshuffled taxa. **(B) **Data exhibiting lack of phylogenetic signal, i.e. random association between CHCs and the phylogeny (see Figure 6B) where the number of parsimony character steps for the reference tree fell within the 95% confidence interval. If the parsimony character steps for the reference tree fell on the extreme right of the distribution (not observed with our data) that would imply that CHC distributions were less conserved than by chance alone (e. g. due to character displacement). Vertical red lines represent 95% confidence intervals and green lines denote the mean number of parsimony character steps for the trees with reshuffled taxa. Red arrows represent the parsimony character steps for the reference tree. Click here for file

Additional file 3 **Figure S3. Gas chromatograms showing representative species-specific CHC profiles of males and females in the *D. buzzatii *species cluster**. Click here for file

Additional file 4 **Table S1. CHC amounts for the 12 major hydrocarbon peaks, out of 36 used to calculate total CHCs (ng/fly), found in the *D. buzzatii *species cluster**. Equivalent chain lengths were used instead of the hydrocarbon names, because of the qualitative differences found among the different species. See Table 2 for the corresponding hydrocarbon names. F = female; M = male. Click here for file

Additional file 5 **Table S2. The first five canonical variates (CVs) based on the total canonical structure of 18 populations/species of the *D. buzzatii *cluster**. CDF analysis included sex as a variable in the model. Values in parentheses represent the percent of total variance explained by each CV. Statistical significance of Pearson correlation coefficients between the original variables and canonical discriminant function loadings are indicated. Click here for file

Additional file 6 **Table S3. The first five canonical variates (CVs) based on the total canonical structure of 14 populations/species of the *D. buzzatii *cluster after deleting the four *D. serido *populations**. CDF analysis included sex as a variable in the model. Values in parentheses represent the percent of total variance explained by each CV. Statistical significance of Pearson correlation coefficients between the original variables and canonical discriminant function loadings are indicated. Click here for file

Additional file 7 **Table S4. Results of the stepwise discriminant analysis based on 18 populations/species of the *D. buzzatii *cluster (see Table **1**)**. The forward elimination method yielded the same results as the backward method, but the latter could not be used because all variables significantly discriminated between populations/species. Click here for file

Additional file 8 **Figure S4. The six most parsimonious trees recovered based on chromosomal inversions and *per *gene sequence data**. Bootstrap support (1,000 replicates and 100 random additions) is shown above the branches. Only bootstrap values above 50% are shown. Click here for file

Additional file 9 **Table S5. Analysis of congruence between the chromosomal inversion plus *per *gene phylogeny and CHC data**. The reconstructed phylogeny used in the character evolution analysis represents the first out of six most parsimonious trees and was based on 13 populations/species of the *D. buzzatii *cluster. The species of the *D. mojavensis *cluster were not included in this analysis. CDF analysis was based on 21 CHC peaks to generate the canonical variates (CVs). LP = linear parsimony; SCPG = squared-change parsimony gradual; and SCPP = squared-change parsimony punctuated. See Table 4 for details. *P*-values in bold represent significant values after false discovery rate (FDR) analysis. See Additional File 12: Table S8 for FDR calculations. Click here for file

Additional file 10 **Table S6. The first five canonical variates based on the total canonical structure of 11 populations/species of the *D. buzzatii *cluster (after deleting the two *D. serido *populations) plus the three species of the *D. mojavensis *cluster used in the phylogenetic reconstruction**. Both sexes were analyzed together in the same CDF analysis to avoid scale effects, but were mapped separately onto the phylogeny (see Figure 6). Values in parentheses represent the percent of the total variance explained by each CV. Statistical significance of Pearson correlation coefficients between the original variables and canonical discriminant function loadings is indicated. Click here for file

Additional file 11 **Table S7. Analysis of congruence between the chromosomal inversion + *per *gene phylogeny and CHC data**. The reconstructed phylogeny used in the character evolution analysis represents the first out of six most parsimonious trees and was based on 11 populations/species of the *D. buzzatii *cluster, after deleting the two *D. serido *populations, plus three species of the *D. mojavensis *cluster. LP = linear parsimony; SCPG = squared-change parsimony gradual; and SCPP = squared-change parsimony punctuated. Besides the three parsimony methods, the detection of phylogenetic signal was also examined with the test for serial independence (TFSI) (run with 1,000 replicates) using the program Phylogenetic Independence 2.0 [69]. See Table 4 for details. *P*-values in bold represent significant values after false discovery rate (FDR) analysis. See Additional File 12: Table S8 for FDR calculations. Click here for file

Additional file 12 **Table S8. False discovery rate (FDR) analyses **[70,71]**of the statistical results from the character reconstruction analyses using the three parsimony methods and the test for serial independence**. FDR analyses were calculated for the three different data sets used to reconstruct the phylogeny: **A) **13 populations/species of the *D. buzzatii *cluster plus the three species of the *D. mojavensis *cluster; **B) **13 populations/species of the *D. buzzatii *cluster (no outgroups); and **C) **11 populations/species of the *D. buzzatii *cluster (no *D. serido *populations) plus the three species of the *D. mojavensis *cluster. For each of these three data sets, significant values after FDR analysis are shown in bold in Table 4, Additional File 9: Table S5 and Additional File 11: Table S7, respectively. FDR analyses were calculated separated for females and males. Probabilities are given in increasing order. Click here for file
