# Supplementary figures and images for: Variations on a theme: diversification of cuticular hydrocarbons in a clade of cactophilic Drosophila
Source: BMC Evol Biol. 2011 Jun 23;11:179. doi: 10.1186/1471-2148-11-179 (PMC3161901; doi:10.1186/1471-2148-11-179)

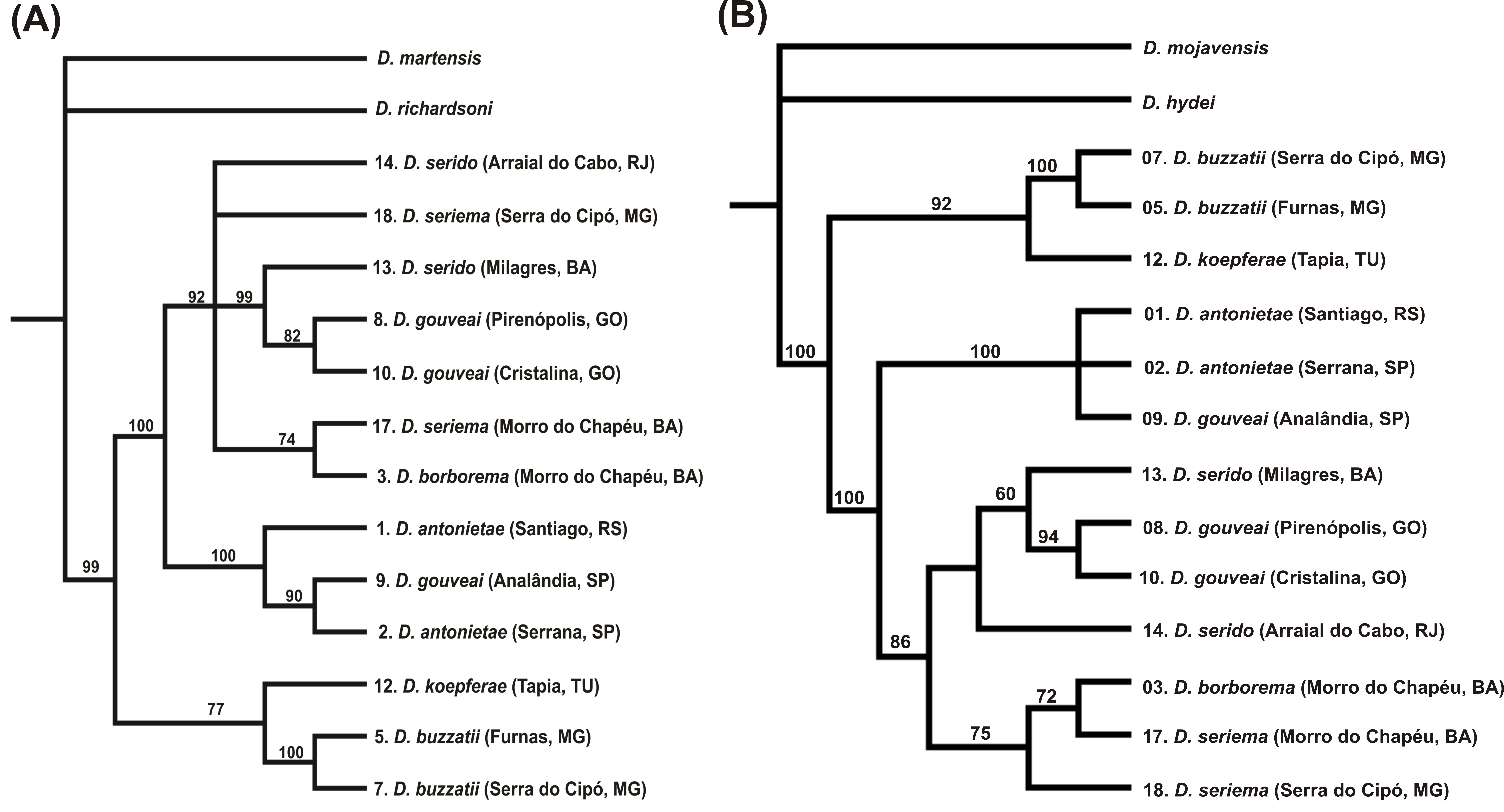

Supplement: Additional file 1 — Figure S1. Strict consensus trees of the D. buzzatii cluster. A) Phylogeny inferred from mtDNA COI data. B) Phylogeny based on chromosomal inversions + mtDNA + period gene. Bootstrap support (1,000 replicates and 100 random additions) is shown above the branches. Only bootstrap values above 50% are shown. See Figure 5 for strict consensus tree inferred from chromosomal inversions + period gene. [file 1471-2148-11-179-S1.JPEG]

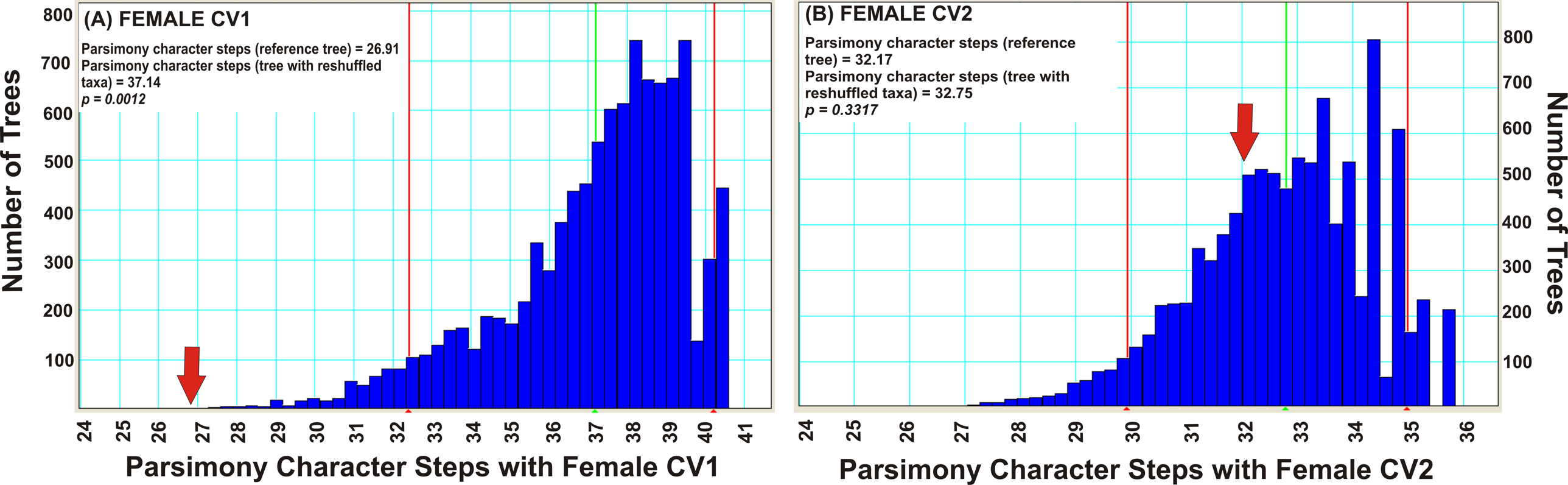

Supplement: Additional file 2 — Figure S2. Bar graphs of random distributions generated by the shuffle option in Mesquite using the Linear Parsimony Method. (A) Data showing presence of phylogenetic signal. The number of parsimony character steps for the reference tree (see Figure 6A) was significantly smaller, i.e. fell on the left side of the distribution, than the number of parsimony character steps for the trees with reshuffled taxa. (B) Data exhibiting lack of phylogenetic signal, i.e. random association between CHCs and the phylogeny (see Figure 6B) where the number of parsimony character steps for the reference tree fell within the 95% confidence interval. If the parsimony character steps for the reference tree fell on the extreme right of the distribution (not observed with our data) that would imply that CHC distributions were less conserved than by chance alone (e. g. due to character displacement). Vertical red lines represent 95% confidence intervals and green lines denote the mean number of parsimony character steps for the trees with reshuffled taxa. Red arrows represent the parsimony character steps for the reference tree. [file 1471-2148-11-179-S2.JPEG]

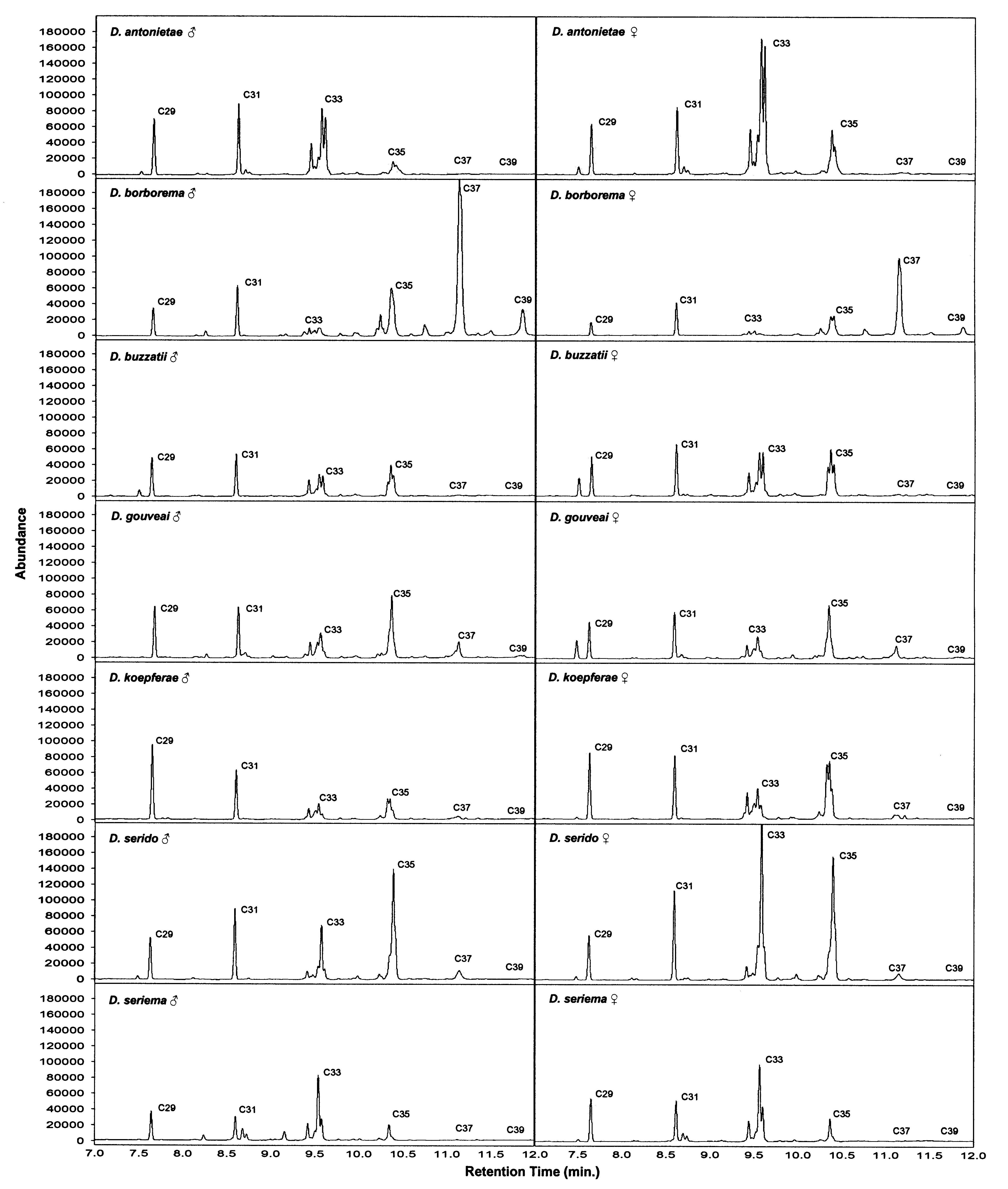

Supplement: Additional file 3 — Figure S3. Gas chromatograms showing representative species-specific CHC profiles of males and females in the D. buzzatii species cluster. [file 1471-2148-11-179-S3.JPEG]

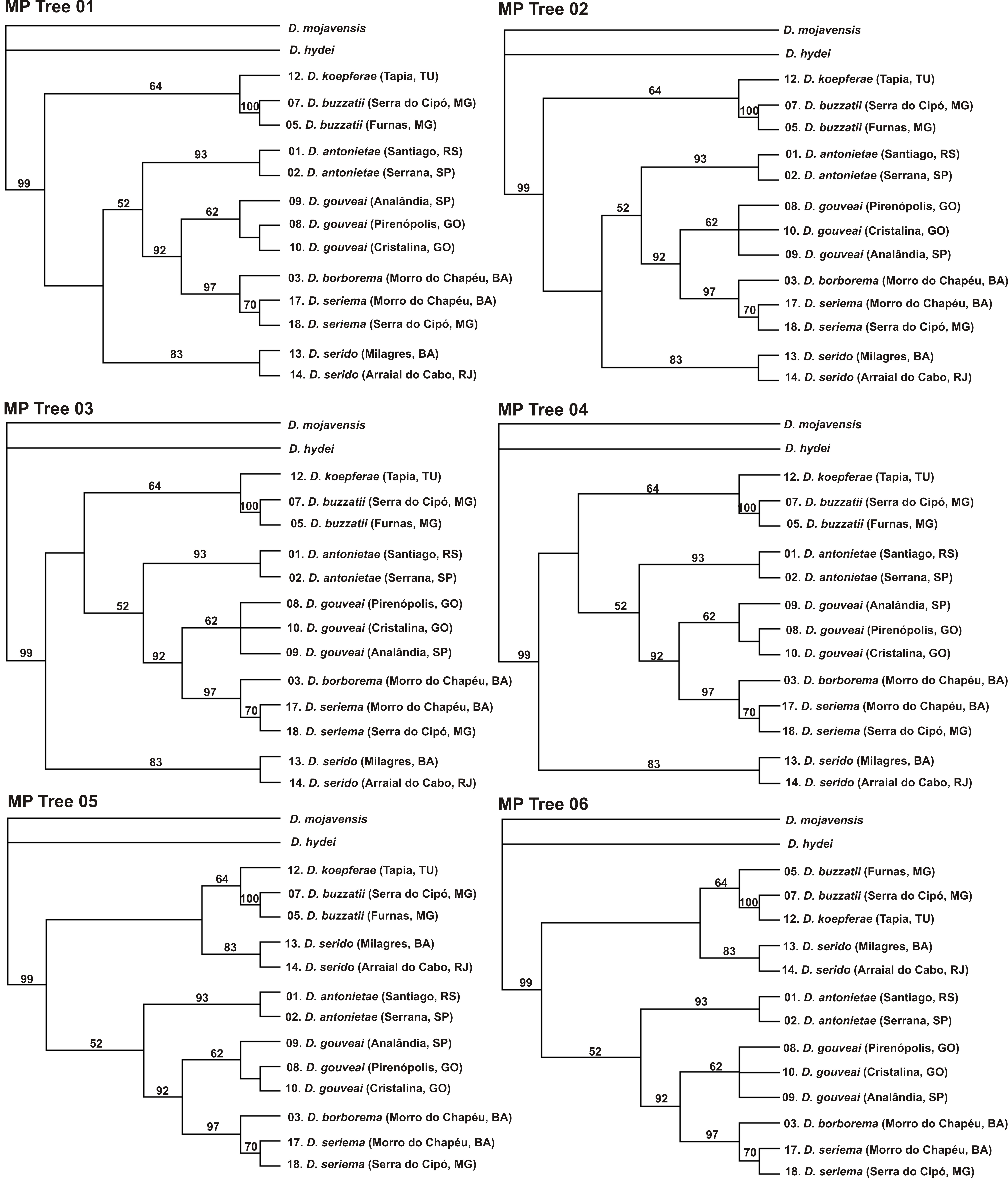

Supplement: Additional file 8 — Figure S4. The six most parsimonious trees recovered based on chromosomal inversions and per gene sequence data. Bootstrap support (1,000 replicates and 100 random additions) is shown above the branches. Only bootstrap values above 50% are shown. [file 1471-2148-11-179-S8.JPEG]
