# Supplementary material for: Variations on a theme: diversification of cuticular hydrocarbons in a clade of cactophilic Drosophila
Source: BMC Evol Biol. 2011 Jun 23;11:179. doi: 10.1186/1471-2148-11-179 (PMC3161901; doi:10.1186/1471-2148-11-179)
Supplement: Additional file 4 — Table S1. CHC amounts for the 12 major hydrocarbon peaks, out of 36 used to calculate total CHCs (ng/fly), found in the D. buzzatii species cluster. Equivalent chain lengths were used instead of the hydrocarbon names, because of the qualitative differences found among the different species. See Table 2 for the corresponding hydrocarbon names. F = female; M = male. [file 1471-2148-11-179-S4.PDF]

Population	Sex	$C_{28.65}$	$C_{30.65}$	$C_{30.83}$	$C_{32.47}$	$C_{32.63}$	$C_{32.70}$	$C_{32.79}$	$C_{34.59}$	$C_{34.66}$	$C_{34.79}$	$C_{36.5}$	$C_{36.7}$	Total CHC Amount
1. *D. antonietae*	F	73.10	90.14	7.77	66.92	47.24	193.33	213.27	10.74	74.54	62.76	2.75	3.66	948.74
(Santiago, RS)	M	78.92	90.17	6.92	76.38	50.55	240.63	225.23	12.90	86.93	66.03	3.24	4.05	1128.77
2. *D. antonietae*	F	144.99	207.15	13.86	128.69	119.38	409.77	326.78	25.44	117.40	94.69	3.24	3.15	1781.30
(Serrana , SP)	M	106.51	124.28	6.87	74.48	56.81	216.78	169.09	10.49	56.68	52.34	1.72	1.45	1100.00
3. *D. borborema*	F	36.60	90.05	0.48	12.41	13.28	8.60	1.81	7.20	64.71	72.33	286.87	146.09	864.64
(Morro do Chapéu, BA)	M	70.86	122.46	0.78	19.17	15.22	23.58	14.19	147.95	54.41	41.30	384.21	264.93	1533.28
4. *D. buzzatti*	F	91.52	115.15	4.11	55.82	39.17	95.07	94.71	62.20	100.55	80.74	4.27	3.33	833.27
(Osório, RS)	M	78.37	89.04	1.63	44.13	22.79	55.30	47.30	31.27	64.33	45.19	1.65	4.34	565.60
5. *D. buzzatti*	F	71.04	103.76	2.86	81.39	50.21	119.70	140.08	90.23	178.71	189.17	8.93	11.35	1175.54
(Furnas, MG)	M	50.03	64.93	1.08	36.07	22.61	48.99	39.80	46.40	76.38	55.48	3.78	4.26	515.82
6. *D. buzzatti*	F	60.68	92.12	2.43	42.94	25.71	65.93	104.08	29.49	87.80	105.12	4.50	5.34	691.29
(Milagres, BA)	M	71.56	138.14	2.97	50.18	22.74	68.58	116.48	32.87	113.50	144.16	0.82	4.59	856.57
7. *D. buzzatti*	F	76.77	122.52	3.64	59.59	50.53	108.10	122.93	80.97	177.26	199.83	7.11	16.18	1138.47
(Serra do Cipó, MG)	M	56.52	101.13	3.44	40.90	29.67	90.11	116.48	45.31	119.29	137.39	3.09	5.64	841.04
8. *D. gouveai*	F	40.01	72.67	3.54	44.27	14.37	80.88	23.00	17.04	270.60	44.05	8.15	74.40	798.96
(Pirenópolis, GO)	M	34.05	51.69	1.29	28.37	9.81	42.87	12.26	15.36	197.48	31.20	6.92	80.39	604.40
9. *D. gouveai*	F	72.01	90.73	0.97	42.81	20.05	56.89	17.43	25.75	295.89	58.85	7.74	91.12	886.18
(Analândia, SP)	M	82.96	94.93	0.62	37.63	16.97	65.04	16.51	32.65	334.92	53.86	11.65	132.72	1010.27
10. *D. gouveai*	F	76.25	114.55	1.32	19.89	27.04	35.24	7.35	23.53	102.96	11.01	7.73	30.82	521.14
(Cristalina, GO)	M	69.72	85.23	1.08	20.74	27.03	43.04	8.57	30.31	132.58	14.31	9.58	38.74	557.94
11. *D. gouveai*	F	94.84	126.14	3.57	37.51	36.82	76.08	17.84	38.00	197.30	23.31	12.46	51.04	841.52
(Ibotirama, BA)	M	107.37	108.48	3.25	34.60	47.15	71.18	9.68	72.80	152.62	14.17	17.73	51.12	852.66
12. *D. koepferae*	F	166.19	174.59	1.96	62.51	67.12	83.14	38.20	139.47	134.49	72.10	9.15	10.11	1094.88
(Tapia, TU)	M	180.73	125.43	3.26	41.31	48.28	53.48	18.24	66.64	68.24	24.31	5.67	17.54	749.90
13. *D. serido*	F	106.50	202.27	5.55	9.61	92.32	421.16	61.99	62.88	355.63	68.87	14.36	10.91	1570.85
(Milagres, BA)	M	102.82	147.96	1.74	5.54	36.44	134.84	21.41	55.97	333.53	54.04	32.30	7.95	1073.61
14. *D. serido*	F	54.10	58.23	239.94	5.00	7.85	45.51	70.93	5.20	8.96	2.62	0.41	0.44	592.53
(Arraial do Cabo, RJ)	M	54.90	58.05	233.62	6.63	8.67	56.46	78.99	8.51	12.86	3.21	0.88	1.12	674.06

15. *D. serido*	F	102.79	135.11	2.45	71.58	44.74	97.42	93.85	76.35	141.96	113.88	6.77	5.91	988.22
(Macaé , RJ)	M	68.91	85.70	2.41	46.91	23.67	70.96	83.85	34.35	96.45	96.29	2.71	5.24	680.63
16. *D. serido*	F	42.16	71.02	248.19	9.11	7.77	51.45	120.99	12.89	26.13	7.51	1.02	1.81	729.45
(Mucuri, BA)	M	41.91	69.46	273.26	9.65	7.05	58.04	115.92	16.60	34.82	12.41	1.92	3.42	911.34
17. *D. seriema*	F	95.21	113.08	14.47	50.65	21.71	197.46	96.38	3.13	58.95	13.20	1.47	0.34	754.02
(Morro do Chapéu, BA)	M	118.82	87.74	26.02	39.32	29.74	167.69	61.44	2.73	40.85	8.97	1.72	0.66	714.89
18. *D. seriema*	F	67.68	70.83	22.62	36.24	14.04	135.63	96.01	5.74	85.25	36.61	2.59	11.59	683.77
(Serra do Cipó, MG)	M	90.11	70.51	40.98	45.85	24.46	197.86	93.55	8.48	101.19	24.13	2.53	10.87	960.80
