# Supplementary material for: Variations on a theme: diversification of cuticular hydrocarbons in a clade of cactophilic Drosophila
Source: BMC Evol Biol. 2011 Jun 23;11:179. doi: 10.1186/1471-2148-11-179 (PMC3161901; doi:10.1186/1471-2148-11-179)
Supplement: Additional file 5 — Table S2. The first five canonical variates (CVs) based on the total canonical structure of 18 populations/species of the D. buzzatii cluster. CDF analysis included sex as a variable in the model. Values in parentheses represent the percent of total variance explained by each CV. Statistical significance of Pearson correlation coefficients between the original variables and canonical discriminant function loadings are indicated. [file 1471-2148-11-179-S5.PDF]

**Carbon Number**	**CHC Peak**	**CV1 (48%)**	**CV2 (20%)**	**CV3 (15%)**	**CV4 (8%)**	**CV5 (3%)**
29	$C_{28.65}$	0.007 ns	-0.219****	0.205***	0.288****	0.153**
31	$C_{30.65}$	0.126*	-0.256****	0.123*	0.135*	-0.059 ns
	$C_{30.78}$	-0.159**	0.612****	-0.504****	0.232****	0.158**
	$C_{30.83}$	0.330****	0.871****	-0.090 ns	0.087 ns	0.147*
33	C_{33br2}	-0.053 ns	-0.677****	-0.437****	-0.159**	-0.099 ns
	C_{33br3}	0.353****	-0.398****	0.346****	0.409****	0.047 ns
	$C_{32.47}$	-0.779****	-0.153**	0.272****	0.252****	0.064 ns
	$C_{32.56}$	0.082 ns	-0.047 ns	0.493****	0.403****	-0.225****
	$C_{32.63}$	-0.085 ns	-0.443****	0.375****	0.394****	0.033 ns
	$C_{32.70}$	0.075 ns	0.077 ns	0.144*	0.770****	0.188**
	$C_{32.79}$	-0.037 ns	0.558****	0.484****	0.447****	0.078 ns
	$C_{32.86}$	0.136*	0.719****	0.475****	-0.144*	0.118*
35	C_{35ene1}	0.261****	-0.680****	-0.420****	-0.177**	-0.088 ns
	C_{35ene2}	-0.337****	-0.603****	-0.264****	-0.057 ns	-0.246****
	C_{35ene3}	-0.019 ns	-0.481****	-0.164**	0.000 ns	-0.188**
	$C_{34.59}$	0.093 ns	-0.591****	0.481****	-0.327****	0.180**
	$C_{34.66}$	-0.062 ns	-0.769****	-0.068 ns	0.225****	0.221****
	$C_{34.79}$	-0.263****	-0.542****	0.488****	0.167**	-0.173**
37	C_{37}	-0.052 ns	-0.709****	0.065 ns	-0.234****	-0.240****
	$C_{36.5}$	0.105 ns	-0.679****	-0.185**	-0.305****	-0.457****
	$C_{36.7}$	-0.141*	-0.647****	-0.401****	-0.422****	-0.076 ns

ns = not significant. * $P \leq 0.05$, ** $P \leq 0.01$, *** $P \leq 0.001$, ****$P \leq 0.0001$.
