# Supplementary material for: Variations on a theme: diversification of cuticular hydrocarbons in a clade of cactophilic Drosophila
Source: BMC Evol Biol. 2011 Jun 23;11:179. doi: 10.1186/1471-2148-11-179 (PMC3161901; doi:10.1186/1471-2148-11-179)
Supplement: Additional file 6 — Table S3. The first five canonical variates (CVs) based on the total canonical structure of 14 populations/species of the D. buzzatii cluster after deleting the four D. serido populations. CDF analysis included sex as a variable in the model. Values in parentheses represent the percent of total variance explained by each CV. Statistical significance of Pearson correlation coefficients between the original variables and canonical discriminant function loadings are indicated. [file 1471-2148-11-179-S6.PDF]

**Carbon Number**	**CHC Peak**	**CV1 (46%)**	**CV2 (27%)**	**CV3 (12%)**	**CV4 (6%)**	**CV5 (4%)**
29	$C_{28.65}$	0.239***	-0.036 ns	-0.067 ns	-0.632****	-0.213**
31	$C_{30.65}$	0.046 ns	-0.104 ns	-0.132*	-0.289****	0.042 ns
	$C_{30.78}$	-0.112 ns	0.777****	0.159*	-0.369****	0.072 ns
	$C_{30.83}$	0.313****	0.728****	-0.065 ns	-0.288****	-0.057 ns
33	C_{33br2}	-0.647****	-0.250****	0.237***	0.033 ns	0.302****
	C_{33br3}	0.411****	-0.145*	-0.084 ns	-0.164*	-0.210**
	$C_{32.47}$	0.601****	0.193**	0.126 ns	-0.077 ns	-0.013 ns
	$C_{32.56}$	0.554****	0.102 ns	-0.303****	0.057 ns	0.210**
	$C_{32.63}$	0.500****	-0.110 ns	-0.040 ns	-0.509****	0.223***
	$C_{32.70}$	0.526****	0.588****	0.133*	-0.210**	0.027 ns
	$C_{32.79}$	0.780****	0.408****	-0.078 ns	0.059 ns	0.044 ns
	$C_{32.86}$	0.795****	-0.015 ns	-0.113 ns	0.031 ns	0.103 ns
35	C_{35ene1}	-0.754****	-0.341****	0.134*	-0.093 ns	0.183**
	C_{35ene2}	-0.669****	-0.185**	-0.145*	0.101 ns	-0.104 ns
	C_{35ene3}	-0.355****	-0.146*	0.001 ns	0.027 ns	0.336****
	$C_{34.59}$	0.157*	-0.811****	0.141*	-0.048 ns	0.032 ns
	$C_{34.66}$	-0.203**	-0.184**	0.614****	0.266****	-0.037 ns
	$C_{34.79}$	0.411****	-0.347****	-0.127 ns	0.512****	0.069 ns
37	C_{37}	-0.302****	-0.617****	-0.133*	0.011 ns	0.285****
	$C_{36.5}$	-0.629****	-0.488****	-0.300****	0.116 ns	0.232***
	$C_{36.7}$	-0.733****	-0.429****	0.189**	0.247***	0.060 ns

ns = not significant. * $P \leq 0.05$, ** $P \leq 0.01$, *** $P \leq 0.001$, ****$P \leq 0.0001$.
