# Supplementary material for: Variations on a theme: diversification of cuticular hydrocarbons in a clade of cactophilic Drosophila
Source: BMC Evol Biol. 2011 Jun 23;11:179. doi: 10.1186/1471-2148-11-179 (PMC3161901; doi:10.1186/1471-2148-11-179)
Supplement: Additional file 7 — Table S4. Results of the stepwise discriminant analysis based on 18 populations/species of the D. buzzatii cluster (see Table 1). The forward elimination method yielded the same results as the backward method, but the latter could not be used because all variables significantly discriminated between populations/species. [file 1471-2148-11-179-S7.PDF]

Number of Steps Entered	Variable Entered	Partial R-Square	F Value	*P*	Wilks' Lambda	*P*	Average Squared Canonical Correlation	*P*
1	$C_{30.83}$	0.905	156.91	< 0.0001	0.095	< 0.0001	0.053	< 0.0001
2	$C_{30.78}$	0.930	217.87	< 0.0001	0.007	< 0.0001	0.104	< 0.0001
3	$C_{32.63}$	0.902	150.30	< 0.0001	0.001	< 0.0001	0.145	< 0.0001
4	$C_{32.47}$	0.915	175.30	< 0.0001	0.000	< 0.0001	0.197	< 0.0001
5	$C_{32.70}$	0.887	127.97	< 0.0001	0.000	< 0.0001	0.243	< 0.0001
6	$C_{34.66}$	0.838	83.55	< 0.0001	0.000	< 0.0001	0.276	< 0.0001
7	$C_{32.56}$	0.777	56.28	< 0.0001	0.000	< 0.0001	0.312	< 0.0001
8	C_{33br3}	0.742	46.26	< 0.0001	0.000	< 0.0001	0.349	< 0.0001
9	$C_{34.59}$	0.731	43.56	< 0.0001	0.000	< 0.0001	0.363	< 0.0001
10	C_{35ene2}	0.664	31.56	< 0.0001	0.000	< 0.0001	0.387	< 0.0001
11	C_{33br2}	0.667	31.84	< 0.0001	0.000	< 0.0001	0.415	< 0.0001
12	$C_{32.79}$	0.607	24.40	< 0.0001	0.000	< 0.0001	0.440	< 0.0001
13	$C_{32.86}$	0.596	23.24	< 0.0001	0.000	< 0.0001	0.459	< 0.0001
14	C_{35ene1}	0.500	15.73	< 0.0001	0.000	< 0.0001	0.479	< 0.0001
15	$C_{28.65}$	0.506	16.00	< 0.0001	0.000	< 0.0001	0.497	< 0.0001
16	$C_{36.5}$	0.418	11.21	< 0.0001	0.000	< 0.0001	0.508	< 0.0001
17	$C_{34.79}$	0.369	9.08	< 0.0001	0.000	< 0.0001	0.520	< 0.0001
18	$C_{36.7}$	0.298	6.55	< 0.0001	0.000	< 0.0001	0.527	< 0.0001
19	C_{37}	0.263	5.49	< 0.0001	0.000	< 0.0001	0.535	< 0.0001
20	C_{35ene3}	0.209	4.05	< 0.0001	0.000	< 0.0001	0.542	< 0.0001
21	$C_{30.65}$	0.149	2.69	0.0004	0.000	< 0.0001	0.546	< 0.0001
