# Supplementary material for: Variations on a theme: diversification of cuticular hydrocarbons in a clade of cactophilic Drosophila
Source: BMC Evol Biol. 2011 Jun 23;11:179. doi: 10.1186/1471-2148-11-179 (PMC3161901; doi:10.1186/1471-2148-11-179)
Supplement: Additional file 9 — Table S5. Analysis of congruence between the chromosomal inversion plus per gene phylogeny and CHC data. The reconstructed phylogeny used in the character evolution analysis represents the first out of six most parsimonious trees and was based on 13 populations/species of the D. buzzatii cluster. The species of the D. mojavensis cluster were not included in this analysis. CDF analysis was based on 21 CHC peaks to generate the canonical variates (CVs). LP = linear parsimony; SCPG = squared-change parsimony gradual; and SCPP = squared-change parsimony punctuated. See Table 4 for details. P-values in bold represent significant values after false discovery rate (FDR) analysis. See Additional File 12: Table S8 for FDR calculations. [file 1471-2148-11-179-S9.PDF]

Characters	**PARSIMONY METHODS**									**TEST FOR SERIAL INDEPENDENCY (TFSI)**	
	**LP**			**SCPG**			**SCPP**				
	Reference Tree	*Random Trees*	*P*	*Reference Tree*	*Random Trees*	*P*	*Reference Tree*	*Random Trees*	*P*	Observed Mean C-Statistics	*P*
Female CV1	19.26	19.70	0.2400	27.05	35.19	0.2580	57.27	62.74	0.2059	0.0292	0.2900
Female CV2	24.17	24.29	0.4242	66.22	78.11	0.4182	173.65	139.48	0.9481	-0.1443	0.0420
Female CV3	19.89	24.17	**0.0070**	29.53	53.94	0.0799	66.64	96.15	0.0357	0.2938	**0.0110**
Female CV4	23.46	26.42	0.0598	20.22	52.43	**0.0092**	60.14	93.54	**0.0055**	0.3446	**0.0070**
Female CV5	21.45	25.95	0.0669	16.65	34.52	0.0373	39.42	61.95	0.0615	0.3083	0.0410
Male CV1	33.09	33.17	0.3807	175.17	167.07	0.5217	277.42	297.91	0.2216	-0.0466	0.2420
Male CV2	20.63	21.57	0.1819	48.41	55.41	0.4236	125.48	98.76	0.9400	-0.1641	0.0550
Male CV3	20.99	23.56	0.0862	30.45	48.56	0.1697	67.29	87.54	0.0873	0.2218	0.0750
Male CV4	18.65	18.45	0.5008	8.53	26.45	**0.0100**	33.90	47.47	0.0509	0.3148	**0.0070**
Male CV5	19.17	27.07	0.0132	14.58	37.69	**0.0133**	34.54	67.27	0.0159	0.3873	**0.0180**
