# Supplementary material for: Variations on a theme: diversification of cuticular hydrocarbons in a clade of cactophilic Drosophila
Source: BMC Evol Biol. 2011 Jun 23;11:179. doi: 10.1186/1471-2148-11-179 (PMC3161901; doi:10.1186/1471-2148-11-179)
Supplement: Additional file 10 — Table S6. The first five canonical variates based on the total canonical structure of 11 populations/species of the D. buzzatii cluster (after deleting the two D. serido populations) plus the three species of the D. mojavensis cluster used in the phylogenetic reconstruction. Both sexes were analyzed together in the same CDF analysis to avoid scale effects, but were mapped separately onto the phylogeny (see Figure 6). Values in parentheses represent the percent of the total variance explained by each CV. Statistical significance of Pearson correlation coefficients between the original variables and canonical discriminant function loadings is indicated. [file 1471-2148-11-179-S10.PDF]

Carbon Number	CHC Peak	CV1 (22%)	CV2 (17%)	CV3 (15%)	CV4 (11%)	CV5 (9%)
29	$C_{28.65}$	-0.425****	-0.576****	-0.086 ns	-0.112*	0.170**
31	$C_{30.65}$	-0.624****	0.361****	0.188***	-0.239****	0.143*
	$C_{30.78}$	-0.272****	-0.005 ns	-0.368****	0.655****	-0.047 ns
	$C_{30.83}$	0.227****	-0.039 ns	-0.525****	0.655****	-0.120*
33	C_{33br2}	-0.695****	0.049 ns	0.377****	0.014 ns	0.064 ns
	C_{33br3}	0.600****	-0.228****	-0.398****	-0.336****	0.249****
	$C_{32.47}$	-0.739****	-0.073 ns	0.065 ns	-0.086 ns	0.086 ns
	$C_{32.56}$	0.014 ns	0.942****	-0.052 ns	-0.170**	0.086 ns
	$C_{32.63}$	-0.360****	-0.432****	-0.095 ns	-0.108 ns	0.107 ns
	$C_{32.70}$	-0.147**	-0.080 ns	-0.567****	0.301****	0.405****
	$C_{32.79}$	0.128*	-0.025 ns	-0.575****	0.183***	0.421****
	$C_{32.86}$	0.494****	-0.088 ns	-0.511****	-0.144**	0.039 ns
35	C_{35ene1}	-0.460****	0.345****	0.352****	-0.020 ns	0.081 ns
	C_{35ene2}	-0.462****	0.323****	0.401****	-0.058 ns	0.054 ns
	C_{35ene3}	0.757****	-0.216****	-0.042 ns	-0.046 ns	0.333****
	$C_{34.59}$	-0.578****	-0.227****	0.198 ***	-0.106 ns	-0.038 ns
	$C_{34.66}$	-0.712****	0.210***	0.257****	-0.121*	-0.045 ns
	$C_{34.79}$	0.599****	-0.130*	-0.246****	-0.183**	-0.267****
37	C_{37}	-0.514****	-0.221****	0.264****	0.031 ns	0.052 ns
	$C_{36.5}$	-0.022 ns	0.111*	0.815****	0.191***	0.120*
	$C_{36.7}$	0.580****	-0.148**	0.668****	0.179**	0.082ns

ns = not significant. * $P \leq 0.05$, ** $P \leq 0.01$, *** $P \leq 0.001$, ****$P \leq 0.0001$.
