# Supplementary material for: Variations on a theme: diversification of cuticular hydrocarbons in a clade of cactophilic Drosophila
Source: BMC Evol Biol. 2011 Jun 23;11:179. doi: 10.1186/1471-2148-11-179 (PMC3161901; doi:10.1186/1471-2148-11-179)
Supplement: Additional file 11 — Table S7. Analysis of congruence between the chromosomal inversion + per gene phylogeny and CHC data. The reconstructed phylogeny used in the character evolution analysis represents the first out of six most parsimonious trees and was based on 11 populations/species of the D. buzzatii cluster, after deleting the two D. serido populations, plus three species of the D. mojavensis cluster. LP = linear parsimony; SCPG = squared-change parsimony gradual; and SCPP = squared-change parsimony punctuated. Besides the three parsimony methods, the detection of phylogenetic signal was also examined with the test for serial independence (TFSI) (run with 1,000 replicates) using the program Phylogenetic Independence 2.0 [69]. See Table 4 for details. P-values in bold represent significant values after false discovery rate (FDR) analysis. See Additional File 12: Table S8 for FDR calculations. [file 1471-2148-11-179-S11.PDF]

	PARSIMONY METHODS									TEST FOR SERIAL INDEPENDENCY (TFSI)	
	**LP**			**SCPG**			**SCPP**				
Characters	*Reference Tree*	*Random Trees*	*P*	*Reference Tree*	*Random Trees*	*P*	*Reference Tree*	*Random Trees*	*P*	Observed Mean C-Statistics	*P*
Female CV1	19.91	31.62	**0.0009**	16.85	52.85	**0.0171**	42.24	109.68	**0.0015**	0.5892	**0.0010**
Female CV2	15.27	16.43	0.1303	5.85	21.31	**0.0255**	43.94	44.09	0.0644	0.0477	0.3220
Female CV3	24.32	30.45	**0.0176**	41.42	45.43	0.4892	71.44	94.50	0.0995	0.1367	0.1630
Female CV4	17.31	24.09	**0.0026**	6.59	31.11	**0.0006**	35.43	64.96	**0.0010**	0.4029	**0.0020**
Female CV5	19.33	26.33	**0.0038**	12.34	40.21	**0.0121**	40.13	83.37	**0.0031**	0.4201	**0.0030**
Male CV1	20.69	25.76	**0.0107**	27.17	39.91	0.2484	47.93	82.31	**0.0050**	0.3556	**0.0120**
Male CV2	17.43	17.60	0.3354	7.37	28.10	0.0513	57.34	58.39	0.5074	0.0651	0.2800
Male CV3	26.38	32.83	**0.0125**	85.70	68.17	0.6794	129.16	137.69	0.3396	-0.0518	0.4000
Male CV4	16.13	25.04	**0.0005**	12.67	40.04	0.0173	31.40	83.04	**0.0013**	0.5247	**0.0030**
Male CV5	19.02	24.98	**0.0077**	18.93	33.44	0.1555	39.78	68.86	**0.0183**	0.3125	**0.0200**
