# Supplementary material for: Variations on a theme: diversification of cuticular hydrocarbons in a clade of cactophilic Drosophila
Source: BMC Evol Biol. 2011 Jun 23;11:179. doi: 10.1186/1471-2148-11-179 (PMC3161901; doi:10.1186/1471-2148-11-179)
Supplement: Additional file 12 — Table S8. False discovery rate (FDR) analyses [70,71]of the statistical results from the character reconstruction analyses using the three parsimony methods and the test for serial independence. FDR analyses were calculated for the three different data sets used to reconstruct the phylogeny: A) 13 populations/species of the D. buzzatii cluster plus the three species of the D. mojavensis cluster; B) 13 populations/species of the D. buzzatii cluster (no outgroups); and C) 11 populations/species of the D. buzzatii cluster (no D. serido populations) plus the three species of the D. mojavensis cluster. For each of these three data sets, significant values after FDR analysis are shown in bold in Table 4, Additional File 9: Table S5 and Additional File 11: Table S7, respectively. FDR analyses were calculated separated for females and males. Probabilities are given in increasing order. [file 1471-2148-11-179-S12.PDF]

A) *D. buzzatii* cluster + *D. mojavensis* Cluster

**Linear Parsimony (LP)**			**Squared Change Parsimony Gradual (SCPG)**			**Squared Change Parsimony Punctuated (SCPP)**			**TEST FOR SERIAL INDEPENDENCY (TFSI)**		
Characters	***P***	***Threshold***	***Characters***	***P***	***Threshold***	***Characters***	***P***	***Threshold***	***Characters***	***P***	***Threshold***
Female CV1	0.0012	0.01	Female CV5	0.0306	0.01	Female CV1	0.0026	0.01	Female CV1	0.0020	0.01
Female CV5	0.0040	0.02	Female CV1	0.0921	0.02	Female CV5	0.0223	0.02	Female CV2	0.0090	0.02
Female CV4	0.0931	0.03	Female CV3	0.1756	0.03	Female CV4	0.0683	0.03	Female CV5	0.0480	0.03
Female CV3	0.1287	0.04	Female CV4	0.3025	0.04	Female CV3	0.1976	0.04	Female CV4	0.2210	0.04
Female CV2	0.3317	0.05	Female CV2	0.7182	0.05	Female CV2	0.9553	0.05	Female CV3	0.2460	0.05
Male CV1	0.0004	0.01	Male CV1	0.0002	0.01	Male CV1	0.0004	0.01	Male CV1	0.0010	0.01
Male CV5	0.0080	0.02	Male CV3	0.2850	0.02	Male CV5	0.0699	0.02	Male CV2	0.0490	0.02
Male CV4	0.1142	0.03	Male CV4	0.4929	0.03	Male CV4	0.1340	0.03	Male CV5	0.1590	0.03
Male CV3	0.2032	0.04	Male CV2	0.6803	0.04	Male CV3	0.2689	0.04	Male CV3	0.2930	0.04
Male CV2	0.4783	0.05	Male CV5	0.8830	0.05	Male CV2	0.9652	0.05	Male CV4	0.3000	0.05

B) *D. buzzatii* cluster (No outgroup)

**Linear Parsimony (LP)**			**Squared Change Parsimony Gradual (SCPG)**			**Squared Change Parsimony Punctuated (SCPP)**			**TEST FOR SERIAL INDEPENDENCY (TFSI)**		
Characters	***P***	***Threshold***	***Characters***	***P***	***Threshold***	***Characters***	***P***	***Threshold***	***Characters***	***P***	***Threshold***
Female CV3	0.0070	0.01	Female CV4	0.0092	0.01	Female CV4	0.0055	0.01	Female CV4	0.0070	0.01
Female CV4	0.0598	0.02	Female CV5	0.0373	0.02	Female CV3	0.0357	0.02	Female CV3	0.0110	0.02
Female CV5	0.0669	0.03	Female CV3	0.0799	0.03	Female CV5	0.0615	0.03	Female CV5	0.0410	0.03
Female CV1	0.2400	0.04	Female CV1	0.2580	0.04	Female CV1	0.2059	0.04	Female CV2	0.0420	0.04
Female CV2	0.4242	0.05	Female CV2	0.4182	0.05	Female CV2	0.9481	0.05	Female CV1	0.2900	0.05
Male CV5	0.0132	0.01	Male CV4	0.0100	0.01	Male CV5	0.0159	0.01	Male CV4	0.0070	0.01
Male CV3	0.0862	0.02	Male CV5	0.0133	0.02	Male CV4	0.0509	0.02	Male CV5	0.0180	0.02
Male CV2	0.1819	0.03	Male CV3	0.1697	0.03	Male CV3	0.0873	0.03	Male CV2	0.0550	0.03
Male CV1	0.3807	0.04	Male CV2	0.4236	0.04	Male CV1	0.2216	0.04	Male CV3	0.0750	0.04
Male CV4	0.5008	0.05	Male CV1	0.5217	0.05	Male CV2	0.9400	0.05	Male CV1	0.2420	0.05

C) *D. buzzatii* cluster (No *D. serido* populations) + *D. mojavensis* Cluster

Linear Parsimony (LP)			Squared Change Parsimony Gradual (SCPG)			Squared Change Parsimony Punctuated (SCPP)			TEST FOR SERIAL INDEPENDENCY (TFSI)		
Characters	***P***	***Threshold***	***Characters***	***P***	***Threshold***	***Characters***	***P***	***Threshold***	***Characters***	***P***	***Threshold***
Female CV1	0.0009	0.01	Female CV4	0.0006	0.01	Female CV4	0.0010	0.01	Female CV1	0.0010	0.01
Female CV4	0.0026	0.02	Female CV5	0.0121	0.02	Female CV1	0.0015	0.02	Female CV4	0.0020	0.02
Female CV5	0.0038	0.03	Female CV1	0.0171	0.03	Female CV5	0.0031	0.03	Female CV5	0.0030	0.03
Female CV3	0.0176	0.04	Female CV2	0.0255	0.04	Female CV2	0.0644	0.04	Female CV3	0.1630	0.04
Female CV2	0.1303	0.05	Female CV3	0.4892	0.05	Female CV3	0.0995	0.05	Female CV2	0.3220	0.05
Male CV4	0.0005	0.01	Male CV4	0.0173	0.01	Male CV4	0.0013	0.01	Male CV4	0.0030	0.01
Male CV5	0.0077	0.02	Male CV2	0.0513	0.02	Male CV1	0.0050	0.02	Male CV1	0.0120	0.02
Male CV1	0.0107	0.03	Male CV5	0.1555	0.03	Male CV5	0.0183	0.03	Male CV5	0.0200	0.03
Male CV3	0.0125	0.04	Male CV1	0.2484	0.04	Male CV3	0.3396	0.04	Male CV2	0.2800	0.04
Male CV2	0.3354	0.05	Male CV3	0.6794	0.05	Male CV2	0.5074	0.05	Male CV3	0.4000	0.05

For all 3 datasets:
Critical probability $\alpha = 0.05$
Number of tests (m) in each family = 5
$\alpha/m = 0.01$
